# A Novel Human Long Noncoding RNA *SCDAL* Promotes Angiogenesis through SNF5‐Mediated GDF6 Expression

**DOI:** 10.1002/advs.202004629

**Published:** 2021-07-28

**Authors:** Rongrong Wu, Wangxing Hu, Huan Chen, Yingchao Wang, Qingju Li, Changchen Xiao, Lin Fan, Zhiwei Zhong, Xiaoying Chen, Kaiqi Lv, Shuhan Zhong, Yanna Shi, Jinghai Chen, Wei Zhu, Jianyi Zhang, Xinyang Hu, Jian'an Wang

**Affiliations:** ^1^ Department of Cardiology Second Affiliated Hospital College of Medicine Zhejiang University Hangzhou 310009 P. R. China; ^2^ Cardiovascular Key Laboratory of Zhejiang Province Hangzhou 310009 P. R. China; ^3^ Key Laboratory of Microbial Technology and Bioinformatics of Zhejiang Province Hangzhou 310012 P. R. China; ^4^ Department of Biomedical Engineering University of Alabama at Birmingham School of Medicine and School of Engineering Birmingham AL 35294 USA

**Keywords:** angiogenesis, growth differentiation factor 6, myocardial infarction, *SCDAL*

## Abstract

Angiogenesis is essential for vascular development. The roles of regulatory long noncoding RNAs (lncRNAs) in mediating angiogenesis remain under‐explored. Human embryonic stem cell‐derived mesenchymal stem cells (hES‐MSCs) are shown to exert more potent cardioprotective effects against cardiac ischemia than human bone marrow‐derived MSCs (hBM‐MSCs), associated with enhanced neovascularization. The purpose of this study is to search for angiogenic lncRNAs enriched in hES‐MSCs, and investigate their roles and mechanisms. AC103746.1 is one of the most highly expressed intergenic lncRNAs detected in hES‐MSCs versus hBM‐MSCs, and named as *SCDAL* (stem cell‐derived angiogenic lncRNA). *SCDAL* knockdown significantly reduce the angiogenic potential and reparative effects of hES‐MSCs in the infarcted hearts, while overexpression of *SCDAL* in either hES‐MSCs or hBM‐MSCs exhibits augmented angiogenesis and cardiac function recovery. Mechanistically, *SCDAL* induces growth differentiation factor 6 (GDF6) expression via direct interaction with SNF5 at GDF6 promoter. Secreted GDF6 promotes endothelial angiogenesis via non‐canonical vascular endothelial growth factor receptor 2 activation. Furthermore, *SCDAL‐*GDF6 is expressed in human endothelial cells, and directly enhances endothelial angiogenesis in vitro and in vivo. Thus, these findings uncover a previously unknown lncRNA‐dependent regulatory circuit for angiogenesis. Targeted intervention of the *SCDAL*‐GDF6 pathway has potential as a therapy for ischemic heart diseases.

## Graphical abstract

Long noncoding RNA (lncRNA) *SCDAL* (stem cell‐derived angiogenic lncRNA) is identified as a critical regulator for angiogenic actions of human embryonic stem cell‐derived mesenchymal stem cells (hES‐MSCs) in ischemic hearts. *SCDAL*initiates growth differentiation factor 6 (GDF6) expression by recruiting SNF5 to GDF6 promoter. Secreted GDF6 promotes endothelial angiogenesis through vascular endothelial growth factor receptor 2 pathway.

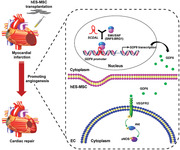

## 1 Introduction

Angiogenesis is essential for tissue development and repair. Aberrant angiogenesis contributes to the pathogenesis of numerous disorders, including cardiovascular diseases, cancer, inflammation, and immune disorders.^[^ ^1^ ^]^ In the process of pathological remodeling in hearts following myocardial infarction (MI), insufficient angiogenesis is a major contributor to trigger transition from compensated hypertrophy to heart failure. Thus, enhancing angiogenesis to restore blood supply has been viewed as a therapeutic approach to salvage myocytes in the ischemic myocardium, in order to preserve function and reduce adverse remodeling. Therefore, understanding the molecular mechanisms of angiogenesis is important to develop new therapies to treat ischemic heart diseases.

Angiogenesis is tightly regulated by coordinated actions of extracellular growth factors, intracellular signaling pathways, and transcription machinery.^[^ ^2^ ^]^ Stem cells exert potent effects on angiogenesis process, potentially by stimulating vessel growth, development and maturation via a broad repertoire of angiogenic paracrine factors. Indeed, human embryonic stem cells (hESCs) and human induced pluripotent stem cells have been utilized as expansible sources to produce mesenchymal stem cells (MSCs) as therapeutic agents for ischemic diseases.^[^ ^3^ ^]^ Interestingly, compared with human bone marrow‐derived MSCs (hBM‐MSCs) or human fetal MSCs, the hPSC‐derived MSCs have superior therapeutic capacity in severe ischemic injuries, associated with more extensive neovascularization and more potent paracrine effects.^[^ ^3a,c^ ^]^ However, the specific molecular mechanisms by which stem cells promote angiogenesis remain largely elusive.

Less than 2% of the human genome encodes protein‐coding genes, yet over 70% of the genome is still transcribed into RNAs.^[^ ^4^ ^]^ Of these, the majority of transcripts are long noncoding RNAs (lncRNAs), defined as noncoding RNAs with more than 200 nucleotides in length. LncRNAs are emerging as important regulators in a wide variety of physiological and pathological processes. The molecular mechanisms involved in lncRNA functions are diverse covering multiple steps in transcriptional to post‐transcriptional processes.^[^ ^5^ ^]^ Accumulating studies have uncovered important roles of lncRNAs in modulating angiogenesis and vascular diseases,^[^ ^6^ ^]^ such as *MANTIS*, *STEEL*, *LncEGFL7OS*, *SNHG12*, and *GUSBP5‐AS* expressed in vascular endothelial cells,^[^ ^7^ ^]^ *SMILR* and *ANRIL* expressed in vascular smooth muscle cells,^[^ ^8^ ^]^ and *VINAS* enriched in aortic intima.^[^ ^9^ ^]^ However, little is known about the involvement of novel lncRNAs in stem cell‐mediated angiogenesis in ischemic heart diseases apart from a recent report suggesting that H19 mediates partially the cardioprotective roles of atorvastatin‐pretreated MSC‐exosomes in promoting angiogenesis.^[^ ^10^ ^]^

In current study, we profiled highly enriched lncRNAs in hESC‐derived MSCs (hES‐MSCs) compared to hBM‐MSCs, and identified a previously unannotated human/primate‐specific lncRNA, named *SCDAL* (stem cell‐derived angiogenic lncRNA), that functioned as a critical regulator for the angiogenic actions of hES‐MSCs through growth differentiation factor 6 (GDF6)‐mediated endothelial vascular endothelial growth factor receptor 2 (VEGFR2) activation. Furthermore, the *SCDAL*‐GDF6 axis directly mediated endothelial angiogenesis. These findings establish a novel lncRNA‐mediated angiogenic paracrine signaling in human stem cells, and demonstrate its potential as a new therapeutic target for ischemic heart diseases.

## 2 Results

### 2.1 Derivation and Characterization of hES‐MSCs

Mesenchymal differentiation of hESCs was achieved based on a Y‐27632‐assisted monolayer culture system^[^ ^11^ ^]^ (Figure S1A, Supporting Information). After 3–5 passages, the differentiated cells displayed homogeneous fibroblastic morphology typical of hBM‐MSCs (Figure S1A, Supporting Information). Flow cytometry analysis confirmed that the differentiated cells expressed MSC‐specific surface antigens including CD29, CD44, CD90, CD105, and CD166, but did not express hematopoietic lineage markers such as CD34, CD45, CD117, and CD133 (Figure S1B, Supporting Information). Meanwhile, they were uniformly positive for human leukocyte antigen (HLA)‐ABC, but negative for HLA‐DR (Figure S1B, Supporting Information). These profiles were similar to that of hBM‐MSCs (Figure S1B, Supporting Information). Quantitative reverse transcription‐polymerase chain reaction (qRT‐PCR) analysis also indicated a marked decrease in the expression of pluripotent‐associated genes *NANOG*, *OCT4*, and *SOX2* in these differentiated cells compared to their parental hESCs (Figure S1C, Supporting Information). Furthermore, the differentiated cells showed comparable osteogenic, adipogenic, and chondrogenic differentiation potential with hBM‐MSCs (Figure S1D, Supporting Information). Collectively, these fibroblastic cells possessed similar characteristic hallmarks of hBM‐MSCs, referred to hereafter as hES‐MSCs.

### 2.2 hES‐MSCs are More Potent than hBM‐MSCs for Promoting Myocardial Recovery and Angiogenesis

We next investigated the reparative capacity of hES‐MSC or hBM‐MSC therapy on cardiac injury in a mouse acute MI model. Left ventricular (LV) ejection fraction (EF) and fractional shortening (FS) obtained by echocardiographic examination, as well as the rate of LV pressure ±dp/dt obtained by hemodynamic recording were significantly greater on day 28 after MI induction in mice treated with intramyocardial injection of hES‐MSCs than in animals that received an equivalent dose of hBM‐MSCs or an equal volume of the delivery vehicle (Figure S2A–E, Supporting Information).

**Figure 1:**
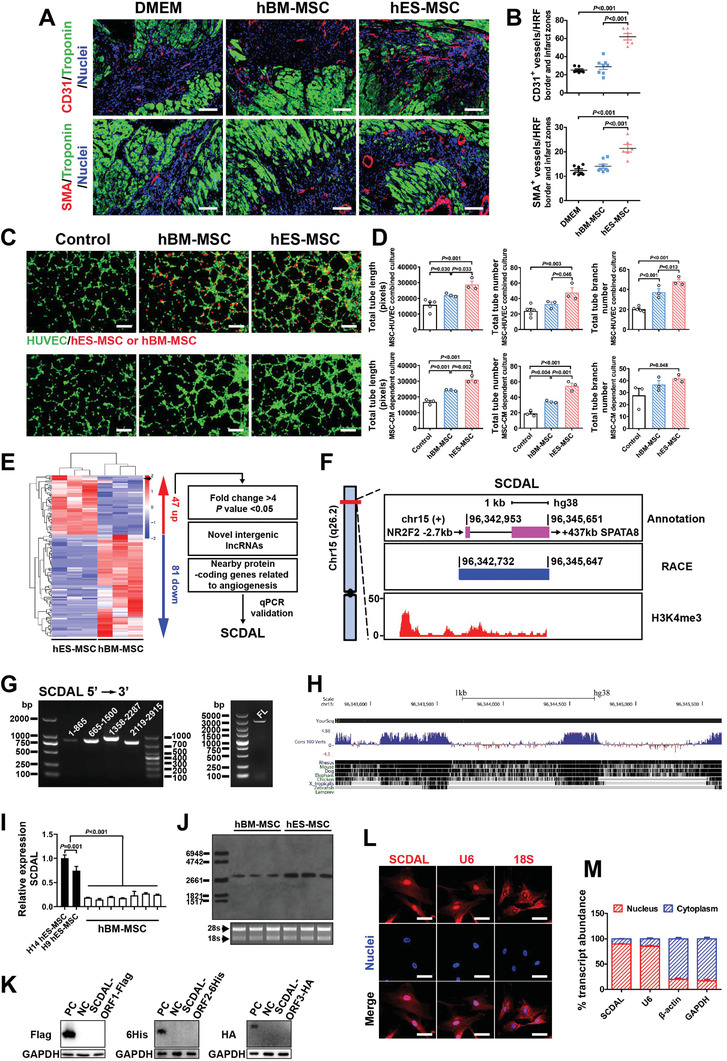
*SCDAL* is highly expressed in the nuclei and may correlate with the angiogenesis of hES‐MSCs. A,B) Representative border zone images to visualize CD31^+^ capillaries and SMA^+^ arterioles four weeks after MI in mice treated with DMEM (*n* = 7), hBM‐MSCs (*n* = 8), and hES‐MSCs (*n* = 6) along with corresponding quantification. Scale bar, 100 µm. HRF, high resolution field. C,D) Representative images and quantification for total tube length, tube number, and tube branch number of HUVECs (GFP expressing, green) cocultured with hBM‐MSCs/hES‐MSCs (PKH26 labeling, red; top panels) or their conditioned supernatants (bottom panels) in comparison with the corresponding control groups (*n* ≥ 3). Scale bar, 200 µm. E) Hierarchical cluster heat map from lncRNA sequencing data (*n* = 3) and selection strategy of *SCDAL*. Black arrow denotes *SCDAL*. F) An aggregated representation of *SCDAL*. Top panel, schematic annotations of *SCDAL* genomic locus on chromosome 15. Purple rectangles represent exons. Numbers indicate genomic position. Middle panel, the genomic locus of full‐length *SCDAL* obtained by RACE. Bottom panel, ChIP‐seq data showing H3K4me3 enrichment at *SCDAL* promoter region. G) RT‐PCR verification of segment and full‐length (FL) of *SCDAL*. H) Sequence conservation analysis of *SCDAL* from the UCSC genome browser. I) qRT‐PCR validation of *SCDAL* expression between hES‐MSCs derived from two hESC lines and hBM‐MSCs of different donors (*n* = 3). J) northern blot confirmation of higher *SCDAL* expression in hES‐MSCs compared to hBM‐MSCs (*n* = 3). K) Western blot detection of fusion proteins from *SCDAL*‐ORFs. Truncated protein‐coding genes fused with different tags are used as positive controls (PC). L) Endogenous *SCDAL* (red) in hES‐MSCs detected by RNA FISH. Probes for U6 (red) and 18S (red) serve as nuclear and cytoplasmic controls, respectively. Scale bar, 10 µm. M) Percentage of nuclear and cytoplasmic RNA concentration of *SCDAL*, *U6* (nuclear marker), *β*‐actin and *GAPDH* (cytoplasmic markers) measured by qRT‐PCR after subcellular fractionation in hES‐MSCs (*n* = 3). All bars in (B), (D), (I), and (M) represent mean ± SEM ((B), (D)，and (I), One‐way ANOVA, LSD, S‐N‐K, and Waller–Duncan analysis).

Associated with the improvements in contractility, the CD31‐positive (CD31^+^) capillary and *α*‐smooth muscle actin‐positive (SMA^+^) arteriole densities in the border and infarct zones were significantly higher in the hES‐MSC treated hearts compared with the hBM‐MSC or vehicle groups at 28 days post MI (**Figure** **1**A,B). Additionally, reduced infarct area and fibrotic area at day 28 post MI (Figure S2F–I, Supporting Information) and decreased cardiac apoptosis (Figure S2J,K, Supporting Information) in the border zone at day 3 post MI were observed in the hES‐MSC treated hearts. We also demonstrated lower levels of CD68‐positive macrophages and CD3‐positive T lymphocytes at the border zones three days after MI in hBM‐MSC and hES‐MSC‐treated hearts compared with MI hearts (Figure S2L–O, Supporting Information), indicating that hBM‐MSCs and hES‐MSCs did not trigger significant immune rejection after administration into xenogeneic animal models.

Increasing evidence indicates that myocardial angio‐ and arteriogenesis are one of major mechanisms responsible for the improvement in LV function with cell therapy after ischemic myocardial injury. To demonstrate that the enhanced angiogenic capacity was directly contributed by hES‐MSCs, we applied two different coculture systems for in vitro Matrigel tube formation assays. First, human umbilical vein endothelial cells (HUVECs) were cocultured with MSCs from different origins. Second, the MSC‐conditioned media (CM) were tested on HUVECs. Both systems confirmed significantly increased total tube length, tube number, and tube branch number of vascular‐like networks in hES‐MSC groups when compared with the corresponding hBM‐MSC and vehicle control groups (Figure 1C,D). The above data suggested that hES‐MSCs had superior angiogenic potential over hBM‐MSCs, most likely via secreted factors.

### 2.3 LncRNA AC103746.1 (*SCDAL*) is Much More Highly Expressed in hES‐MSCs than in hBM‐MSCs

Accumulating reports have shown that the angiogenic activity of vascular cells is partially regulated by lncRNAs;^[^ ^7^, ^8^ ^]^ thus, we investigated whether the elevated angiogenic potency observed in hES‐MSCs may also be attributable to a change in lncRNA expression. From RNA sequencing datasets obtained from hES‐MSCs compared to hBM‐MSCs, we identified 47 up‐regulated and 81 down‐regulated transcripts annotated as lncRNAs with significant expression levels (fold change >2, *p* value < 0.05, Figure 1E). Considering that lncRNAs exert their function largely through *cis*‐regulation of neighboring genes or *trans*‐regulation mechanisms,^[^ ^5a^ ^]^ we compiled a list of novel intergenic lncRNAs which were highly enriched in hES‐MSCs (fold change > 4, *p* value < 0.05) with their genomic loci located near protein‐coding genes with known functions in angiogenesis (Figure 1E). Among those lncRNAs, one of the most highly expressed intergenic lncRNAs in hES‐MSCs is a previously uncharacterized lncRNA AC103746.1 (transcript symbol *Ensembl* version: ENST00000617440.1) that we name it as *SCDAL*. According to the annotations in the *Ensembl* and UCSC Genome Browser (GRCh38/hg38), the human *SCDAL* locus is located on chromosome 15 q26.2, and composed of two exons which spans nearly 2.7 kilobases (kb) (Figure 1F). The nearby protein‐coding gene of *SCDAL* is the nuclear receptor subfamily 2 group F member 2 (NR2F2, also known as COUP‐TFII) gene, which serves as a known regulator in tumor angiogenesis^[^ ^12^ ^]^ (Figure 1F). Publicly available chromatin immuneprecipitation (ChIP)‐seq data from MSC‐differentiated cells confirmed that the transcriptional start site of *SCDAL* was marked by trimethylation of histone H3 at lysine 4 (H3K4me3) (Figure 1F). Rapid amplification of cDNA ends assay demonstrated *SCDAL* to be a 2943 nt polyadenylated RNA with only one exon (Figure 1F,G). The full‐length *SCDAL* is well conserved with nonhuman primate, but only partially conserved beyond other mammalian species (Figure 1H). Confirming the results from RNA sequencing, *SCDAL* expression was detected to be much higher in hES‐MSCs than in different donors of hBM‐MSCs measured by qRT‐PCR (Figure 1I). Northern blot analysis also demonstrated only one abundantly expressed transcript of *SCDAL* in hES‐MSCs relative to hBM‐MSCs, with a length near 3 kb (Figure 1J).

Based on sequence analysis from three bioinformatic programs, including Phylogenetic Codon Substitution Frequency,^[^ ^13^ ^]^ Coding‐Potential Assessment Tool,^[^ ^14^ ^]^ and Coding Potential Calculator,^[^ ^15^ ^]^ *SCDAL* sequence exhibited no protein‐coding potential (Figure S3A–C, Supporting Information). Recent studies have shown that many lncRNAs are translated to micropeptides, even though they are annotated as “noncoding” genes.^[^ ^16^ ^]^ To investigate the peptide‐coding potential from *SCDAL* open reading frames (ORFs) predicated by NCBI ORFfinder (Figure S3D, Supporting Information), a series of constructs were generated in which the Flag, 6His, and HA tags were respectively fused to the C terminus of three most potentially translated *SCDAL* ORFs (Figure S3E, Supporting Information). Western blot analysis using anti‐Flag, ‐6His, and ‐HA antibodies confirmed no obvious production of the expected fusion proteins from these *SCDAL*‐ORFs (Figure 1K), further supporting that *SCDAL* was a noncoding transcript.

RNA fluorescent in situ hybridization (FISH) detected *SCDAL* predominantly in the nuclei of hES‐MSCs, similar to the localization pattern of U6 (Figure 1L). This subcellular localization was further supported by a nucleocytoplasmic fractionation assay from hES‐MSCs (Figure 1M). Thus, the potential biological activity of *SCDAL* likely occurred via regulation of gene transcription, rather than post‐transcriptional processing of gene products.^[^ ^17^ ^]^

### 2.4 The Enhanced Potency of hES‐MSCs for Promoting Angiogenesis and Myocardial Recovery is Dependent on *SCDAL* Expression

**Figure 2:**
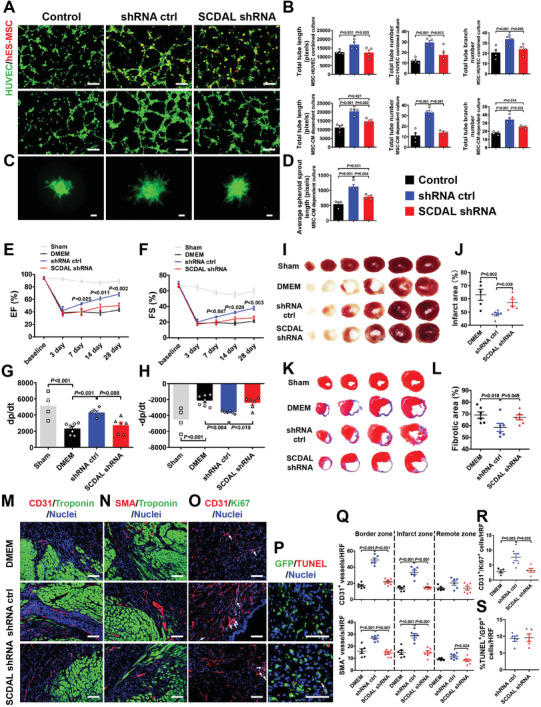
*SCDAL* is required for the angiogenic potential of hES‐MSCs. A,B) Representative images and quantification for total tube length, tube number, and tube branch number of HUVECs (green) cocultured with *SCDAL* shRNA hES‐MSCs (red, top panels) or their conditioned supernatants (middle panels) as compared to control groups (*n* = 4). Scale bar, 200 µm. C,D) Representative images and quantification for average outgrowth length of HUVEC spheroids cocultured with the conditioned supernatants of *SCDAL* shRNA hES‐MSCs as compared to control groups (*n* ≥ 3). Scale bar, 200 µm. E–H) Cardiac functional parameters EF, FS, and ±dp/dt derived from echocardiography and hemodynamics in Sham (*n* ≥ 4), DMEM (*n* = 7), shRNA ctrl hES‐MSC (*n* = 6), and *SCDAL* shRNA hES‐MSC (*n* = 6)‐receiving mice. I–L) Infarct area and fibrotic area quantification respectively by triphenyltetrazolium chloride (TTC) staining and Masson's trichrome staining on serial heart sections four weeks post MI in Sham, DMEM (*n* ≥ 5), shRNA ctrl hES‐MSC (*n* = 6), and *SCDAL* shRNA hES‐MSC (*n* ≥ 5)‐receiving mice. M,N,Q) Representative border zone images and quantification for CD31^+^ capillaries and SMA^+^ arterioles in DMEM (*n* = 6), shRNA ctrl hES‐MSC (*n* = 6), and *SCDAL* shRNA hES‐MSC (*n* = 7)‐receiving mice 28 days after MI. Scale bar, 100 µm. O,R) Representative images and quantification of colocalizing CD31^+^ with Ki67^+^ cells (white arrows) in DMEM (*n* = 4), shRNA ctrl hES‐MSC (*n* = 6), and *SCDAL* shRNA hES‐MSC (*n* = 5)‐treated hearts three days after MI. Scale bar, 100 µm. P,S) Representative images and quantification of colocalizing TUNEL^+^ with GFP^+^ cells (white arrows) in different hES‐MSC (*n* = 5)‐treated hearts three days after transplantation. Scale bar, 100 µm. HRF, high resolution field. All bars in (B), (D–H), (J), (L), and (Q–S) represent mean ± SEM ((B), (D–H), (J), (L), and (Q–R), One‐way ANOVA, LSD, S‐N‐K, and Waller–Duncan analysis; (S), unpaired Student's *t*‐test).

To investigate the potential role of *SCDAL* in hES‐MSC‐induced angiogenesis, loss‐ and gain‐of‐function approaches were used. For this purpose, we constructed lentiviruses expressing either full‐length *SCDAL* or short hairpin RNA (shRNA) targeting *SCDAL*. First, we generated *SCDAL* shRNA hES‐MSCs with ≈50% decrease in expression (Figure S4A, Supporting Information) and confirmed decreased *SCDAL* expression in both nucleus and cytoplasm of *SCDAL* shRNA hES‐MSCs as compared to hES‐MSCs harboring a non‐targeting shRNA (shRNA ctrl) by FISH (Figure S4B, Supporting Information). *SCDAL* shRNA did not affect cell viability and proliferation of hES‐MSCs (Figure S4C,D, Supporting Information). The in vitro tube formation assays showed that HUVECs cocultured with *SCDAL* shRNA hES‐MSCs or their CM exhibited a significant decrease in tube formation on Matrigel, as compared with those from shRNA ctrl hES‐MSCs (**Figure** **2**A,B). The spheroid‐based angiogenesis assay is another sensitive and versatile method to study the impact of pro‐ or anti‐angiogenic determinants on proliferation and sprouting of endothelial cells.^[^ ^7^, ^18^ ^]^ We found that less capillary‐like sprouts were originating from the HUVEC spheroids in response to the CM from *SCDAL* shRNA hES‐MSCs as compared to those from shRNA ctrl hES‐MSC group (Figure 2C,D). To support the specificity of *SCDAL* silencing, we also used a lncRNA Smart Silencer, a mixture of three small interfering RNAs (siRNAs) and three antisense oligonucleotides targeting different regions of *SCDAL* transcript, to deplete *SCDAL* in both nucleus and cytoplasm. As indicated, Smart Silencer achieved more effective knockdown efficiency (≈70% decrease, Figure S4E, Supporting Information). Consistently, *SCDAL* depletion with Smart Silencer did not affect cell viability and proliferation of hES‐MSCs (Figure S4F,G, Supporting Information), but dramatically impaired their angiogenic capacity on HUVECs (Figure S4H,I, Supporting Information).

Consistent with the observation in vitro, transplantation of *SCDAL* shRNA hES‐MSCs resulted in a marked decrease in cardiac functional recovery after MI, as evidenced by attenuated myocardial function (EF, FS, and ±dp/dt; Figure 2E–H; Figure S5A, Supporting Information), greater infarct area and fibrotic area (Figure 2I–L), as well as lower CD31^+^ capillary and SMA^+^ arteriole densities in both border and infarct areas of the infarcted hearts four weeks post infarction (Figure 2M,N,Q; Figure S5B, Supporting Information), as compared with shRNA ctrl hES‐MSC transplantation. Furthermore, we found fewer CD31^+^/Ki67‐positive (Ki67^+^) cells in the *SCDAL* shRNA hES‐MSC‐treated hearts three days after MI compared with control hES‐MSC‐treated hearts (Figure 2O,R). Since lentiviruses targeting *SCDAL* carried the green fluorescent protein (GFP) reporter gene, then we evaluated the apoptosis of hES‐MSCs in mouse hearts three days after transplantation by co‐staining of GFP with TdT‐mediated dUTP Nick‐End Labeling, and found that *SCDAL* knockdown did not affect the survival of transplanted hES‐MSCs (Figure 2P,S).

**Figure 3:**
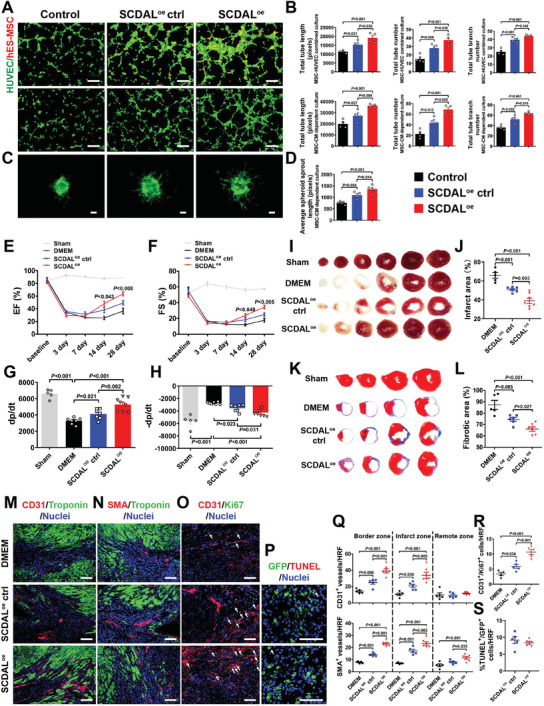
*SCDAL* overexpression enhances the angiogenic capacity of hES‐MSCs. A,B) Representative images and quantification for total tube length, tube number, and tube branch number of HUVEC (green) tube formation after cocultured with *SCDAL* ^oe^ hES‐MSCs (red, top panels) or their conditioned supernatants (middle panels) as compared to control groups (*n* = 4). Scale bar, 200 µm. C,D) Representative images and quantification for average outgrowth length of HUVEC spheroids cocultured with *SCDAL* ^oe^ hES‐MSC‐conditioned supernatants as compared to control groups (*n* ≥ 4). Scale bar, 200 µm. E–H) Cardiac functional parameters EF, FS, and ±dp/dt by echocardiographical and hemodynamic measurements in Sham (*n* = 5), DMEM (*n* = 7), *SCDAL* ^oe^ ctrl hES‐MSC (*n* = 7), and *SCDAL* ^oe^ hES‐MSC (*n* = 8)‐receiving mice. I–L) Infarct area and fibrotic area quantification respectively by TTC staining and Masson's trichrome staining on serial heart sections four weeks post MI in Sham, DMEM (*n* = 5), *SCDAL* ^oe^ ctrl hES‐MSC (*n* = 6), and *SCDAL* ^oe^ hES‐MSC (*n* ≥ 6)‐receiving mice. M,N,Q) Representative border zone images and quantification for CD31^+^ and SMA^+^ vessels in DMEM (*n* = 5), *SCDAL* ^oe^ ctrl hES‐MSC (*n* = 5), and *SCDAL* ^oe^ hES‐MSC (*n* = 6)‐receiving mice 28 days after MI. Scale bar, 100 µm. O,R) Representative images and quantification for CD31^+^/Ki67^+^ cells (white arrows) in DMEM (*n* = 4), *SCDAL* ^oe^ ctrl hES‐MSC (*n* = 5), and *SCDAL* ^oe^ hES‐MSC (*n* = 5)‐treated hearts three days after MI. Scale bar, 100 µm. P,S) Representative images and quantification for TUNEL^+^/GFP^+^ cells (white arrows) in different hES‐MSC (*n* = 5)‐treated hearts three days after transplantation. Scale bar, 100 µm. HRF, high resolution field. All bars in (B), (D–H), (J), (L), and (Q–S) represent mean ± SEM ((B), (D–H), (J), (L), and (Q–R), One‐way ANOVA, LSD, S‐N‐K, and Waller–Duncan analysis; (S), unpaired Student's *t*‐test).

Next, we overexpressed the full‐length *SCDAL* in hES‐MSCs (*SCDAL* ^oe^) to achieve near 60‐fold increase in *SCDAL* level (Figure S6A, Supporting Information). *SCDAL* ^oe^ did not affect cell viability and proliferation of hES‐MSCs (Figure S6B,C, Supporting Information). Enhanced tube formation of HUVECs was observed after cocultured with *SCDAL* ^oe^ hES‐MSCs or the CM from *SCDAL* ^oe^ hES‐MSCs relative to controls (**Figure** **3**A,B). Also, *SCDAL* ^oe^ hES‐MSC supernatants significantly elicited angiogenic sprouting compared to controls (Figure 3C,D). Accordingly, transplantation of *SCDAL* ^oe^ hES‐MSCs led to more improved cardiac function (EF, FS, and ±dp/dt; Figure 3E–H; Figure S6D, Supporting Information) compared with control hES‐MSCs, accompanied by reduced infarct area and fibrotic area (Figure 3I–L) and enhanced angiogenesis in both border and infarct areas four weeks after MI (Figure 3M,N,Q; Figure S6E, Supporting Information). Similarly, enhanced endothelial proliferation as evidenced by increased CD31^+^/Ki67^+^ cells was observed in the hearts treated with *SCDAL* ^oe^ hES‐MSCs compared with controls (Figure 3O,R). Also, *SCDAL* ^oe^ did not affect the survival of transplanted hES‐MSCs in the infarcted myocardium (Figure 3P,S).

Taken together, these results suggested an important role for *SCDAL* in mediating angiogenesis.

### 2.5 *SCDAL* Promotes the Angiogenic Capacity of hES‐MSCs through GDF6

**Figure 4:**
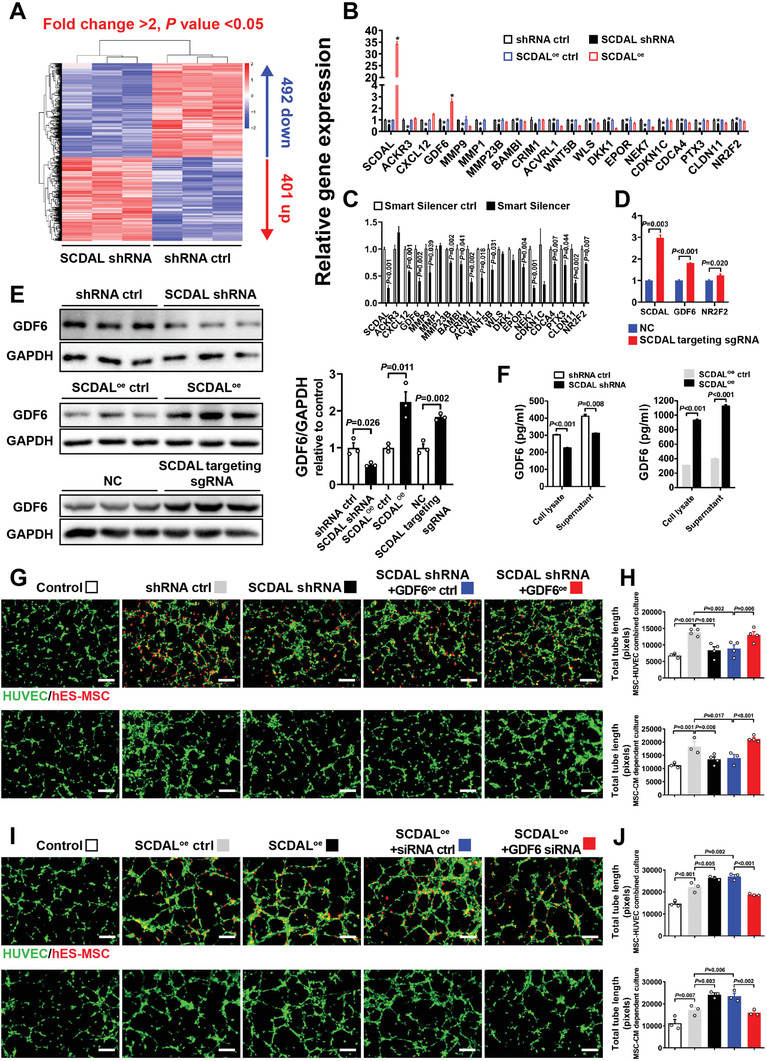
*SCDAL* drives the angiogenic capacity of hES‐MSCs through GDF6. A) Hierarchical clustering of differentially expressed genes in *SCDAL* shRNA hES‐MSCs compared with shRNA ctrl hES‐MSCs as assessed by RNA sequencing (*n* = 3). B) Expression of angiogenesis‐related genes measured by qRT‐PCR in hES‐MSCs after *SCDAL* depletion or overexpression as compared to corresponding controls (*n* ≥ 3). *p < 0.05 versus respective controls. C) Expression of angiogenesis‐related genes in hES‐MSCs after Smart Silencer‐mediated *SCDAL* depletion as compared to control (*n* = 3). D) GDF6 and NR2F2 expression in hES‐MSCs after CRISPR/Cas9‐mediated *SCDAL* induction [*SCDAL* targeting single guide RNA (sgRNA)] as compared to control (*n* = 3). E) Quantification of GDF6 protein levels in hES‐MSCs after *SCDAL* depletion or overexpression or CRISPR/Cas9‐mediated *SCDAL* activation by western blot detection (*n* = 3). F) ELISA examination of GDF6 expression in hES‐MSCs after *SCDAL* depletion or overexpression (*n* = 3). G,H) hES‐MSCs are transfected with shRNA for *SCDAL* knockdown, followed by GDF6 overexpression (GDF6^oe^), and I,J) hES‐MSCs are overexpressed with *SCDAL*, followed by GDF6 knockdown with siRNA (GDF6 siRNA). Representative images (G,I) and quantification (H,J) for total tube length formation of HUVECs (green) cocultured with indicated hES‐MSCs (red) or their supernatants (*n* ≥ 3). All bars in (B–F), (H), and (J) represent mean ± SEM ((B–F), unpaired Student's *t*‐test; (H) and (J), One‐way ANOVA, LSD, S‐N‐K, and Waller–Duncan analysis).

To explore the molecular mechanism underlying the angiogenic function of *SCDAL*, we examined differentially expressed genes in hES‐MSCs after *SCDAL* knockdown. A total of 401 up‐regulated and 492 down‐regulated genes were identified with fold change >2 and adjusted *p* value < 0.05 in the *SCDAL*‐silenced hES‐MSCs compared with the control cells (**Figure** **4**A). Gene Ontology analysis of the differentially expressed genes revealed that the key cellular processes involved in angiogenesis were significantly enriched, such as tube morphogenesis, artery development, and growth factor binding (Figure S7A,B, Supporting Information). Among the genes suppressed by *SCDAL* silencing with either shRNA or Smart Silencer, including the SDF‐1/CXCR7 axis (CXCL12 and ACKR3), GDFs, matrix metallopeptidases, bone morphogenetic protein (BAMBI, CRIM1, and ACVRL1), and WNT (WNT5B, WLS, and DKK1) signaling pathways, erythropoietin receptor, proliferation and survival‐related genes (NEK7, CDKN1C, and CDCA4), as well as other angiogenesis mediators (PTX3 and CLDN11), only GDF6 was significantly up‐regulated upon *SCDAL* overexpression (Figure 4B,C). Also, CRISPR (clustered regularly interspaced short palindromic repeats)/Cas9‐mediated *SCDAL* activation^[^ ^19^ ^]^ significantly up‐regulated GDF6 expression in hES‐MSCs (Figure 4D). However, the adjacent coding gene NR2F2 of *SCDAL* was not as significantly affected as GDF6 upon *SCDAL* overexpression in hES‐MSCs (Figure 4B,D), indicating that *SCDAL* might tend to function as an important *trans*‐acting modulator of GDF6 expression.

Consistence with its changes at mRNA level, the GDF6 protein was also attenuated by *SCDAL* knockdown but increased by *SCDAL* overexpression and CRISPR/Cas9‐mediated *SCDAL* activation (Figure 4E). Additionally, *SCDAL* silencing or overexpression in hES‐MSCs decreased or increased the expression and secretion of GDF6 respectively in both cell lysates and CM detected by enzyme‐linked immunosorbent assay (ELISA) (Figure 4F). Notably, GDF6 expression level was positively correlated with *SCDAL* expression with significantly higher level in hES‐MSCs versus hBM‐MSCs (Figure S7C, Supporting Information). These results suggested that *SCDAL* might serve as an upstream regulator of GDF6.

To determine whether GDF6 is a downstream effector of *SCDAL* in regulating the angiogenic ability of hES‐MSCs, we constructed GDF6 overexpressing lentiviruses and GDF6 siRNA, and found that gene manipulation of GDF6 had no effects on cell viability and proliferation of hES‐MSCs (Figure S7D–G, Supporting Information). First, we re‐expressed GDF6 in *SCDAL*‐silenced hES‐MSCs and implemented in vitro tube formation assays. GDF6 rescue remarkably neutralized the impaired angiogenic ability of hES‐MSCs on HUVECs induced by *SCDAL* knockdown (Figure 4G,H; Figure S7H–K, Supporting Information). Next, we silenced GDF6 in *SCDAL* overexpressing hES‐MSCs and repeated the tube formation assays. GDF6 depletion abolished the enhanced angiogenic potential of *SCDAL* overexpression hES‐MSCs on HUVECs (Figure 4I,J; Figure S7L–O, Supporting Information). These results consistently supported that *SCDAL* promoted the angiogenic capacity of hES‐MSCs in a GDF6‐dependent manner.

### 2.6 *SCDAL*‐Mediated GDF6 Secretion from hES‐MSCs Activates Non‐Canonical VEGFR2 Signaling on Endothelial Cells

**Figure 5:**
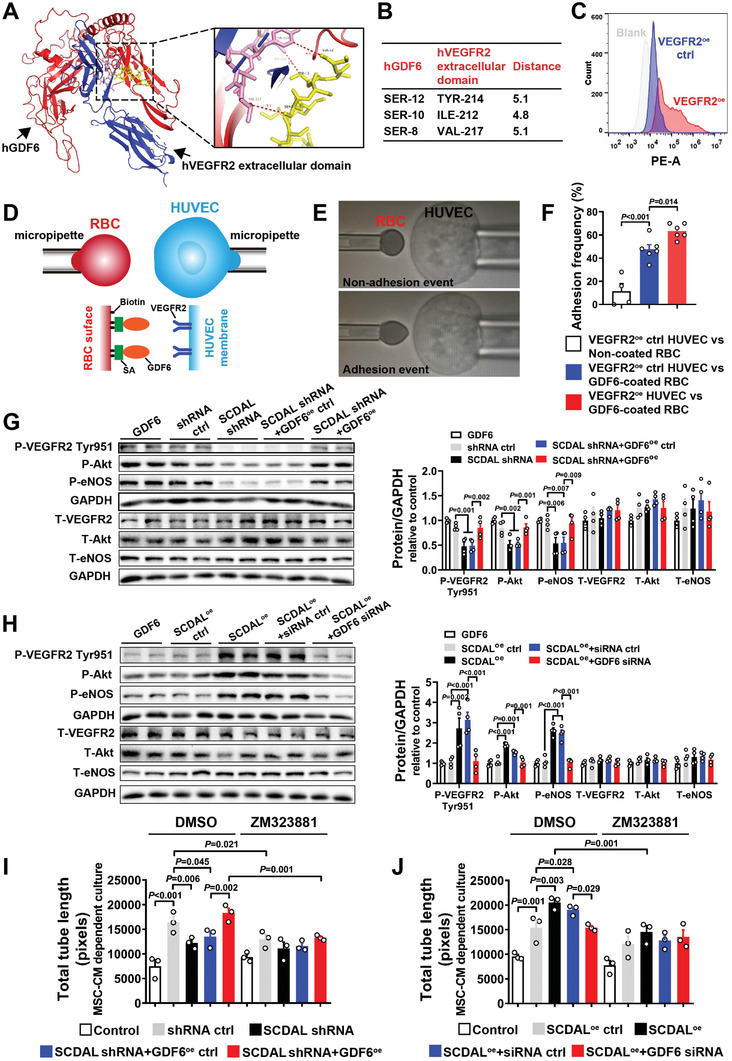
*SCDAL*‐mediated GDF6 secretion activates non‐canonical VEGFR2 signaling on endothelial cells. A) Interface between hGDF6 (red) and hVEGFR2 extracellular domain (blue). The boxed region shows the binding sites with hGDF6 colored in yellow and hVEGFR2 extracellular domain colored in pink. B) The table shows critical interacting residues between hGDF6 and hVEGFR2 extracellular domain. C) Flow cytometry analysis of VEGFR2^+^ cells in VEGFR2 overexpressing HUVECs (VEGFR2^oe^, red) and control HUVECs (VEGFR2^oe^ ctrl, blue). D) Schematics of adhesion frequency assay. A VEGFR2 expressed HUVEC is aspirated by a micropipette (right) and aligned with a GDF6‐coated RBC via biotin‐streptavidin (SA) coupling held by another micropipette (left). E) Schematics of non‐adhesion event and adhesion event. F) Adhesion frequencies between VEGFR2^oe^ ctrl HUVEC and biotinylated RBC without coating, VEGFR2^oe^ ctrl HUVEC and GDF6‐coated RBC or VEGFR2^oe^ HUVEC and GDF6‐coated RBC (*n* ≥ 4). G,H) Quantification of VEGFR2/Akt/eNOS phosphorylations in HUVECs treated with the indicated hES‐MSC supernatants following western blot detection (*n* = 4). Recombinant human GDF6 protein is used as a positive control group. I,J) Quantification of total tube length for HUVEC tube formation after incubation with the indicated hES‐MSC supernatants with or without VEGFR2 blockade (ZM323881) (*n* = 3). DMSO serves as a solvent control. All bars in (F–J) represent mean ± SEM (One‐way ANOVA, LSD, S‐N‐K, and Waller–Duncan analysis).

VEGFR2 is the most prominent activator of angiogenesis in endothelial cells, and can be stimulated by VEGF and other non‐canonical ligands.^[^ ^20^ ^]^ The extracellular domain of VEGFR2 consists of seven immunoglobulin (Ig)‐like domains. Ligand‐induced VEGFR2 activation depend on direct ligand binding to extracellular domains 2 and 3 of receptor.^[^ ^21^ ^]^ By using protein docking predication, we found that the amino acids SER‐12, SER‐10, and SER‐8 of human GDF6 (hGDF6) and the amino acids TYR‐214, ILE‐212, and VAL‐217 of human VEGFR2 (hVEGFR2) extracellular domain were the most potential binding interface between the two proteins, indicating that hGDF6 can function as a novel non‐canonical ligand for hVEGFR2 (**Figure** **5**A,B). Moreover, hGDF6 and mouse VEGFR2 (mVEGFR2) extracellular domain, as well as mouse GDF6 and mVEGFR2 extracellular domain had putative binding sites (Figure S8A–D, Supporting Information). These binding sites were partially conserved and mainly located in VEGFR2 extracellular domains 2 (Figure S8E, Supporting Information).

To explore the binding specificity of GDF6 and VEGFR2 extracellular domain, we used the adhesion frequency assay,^[^ ^22^ ^]^ which has been used to measure two‐dimensional receptor‐ligand binding kinetics. Before assay, flow cytometry analysis confirmed surface expression of VEGFR2 in VEGFR2 overexpressing (VEGFR2^oe^) HUVECs (Figure 5C). We demonstrated that the adhesion frequency of VEGFR2^oe^ HUVECs and GDF6‐coated red blood cells (RBCs) was significantly higher than that of VEGFR2^oe^ ctrl HUVECs and GDF6‐coated RBCs, as well as VEGFR2^oe^ ctrl HUVECs and biotinylated RBCs without coating, indicating a strong interaction of GDF6 and VEGFR2 (Figure 5D–F).

To evaluate the effects of *SCDAL*‐mediated GDF6 secretion on VEGFR2 activation in endothelial cells, we treated HUVECs with the conditioned supernatants from hES‐MSCs (Figure S9A, Supporting Information). Western blot and ELISA (Figure S9B–E, Supporting Information) confirmed intra‐ and extracellular GDF6 protein levels in different hES‐MSC groups. In HUVECs treated with CM from *SCDAL*‐silencing hES‐MSCs, VEGFR2 phosphorylation at Tyr951 was reduced, accompanied by attenuated activation of Akt and endothelial nitric synthase (eNOS) signaling compared to HUVECs stimulated with control hES‐MSC supernatants. The impaired VEGFR2/Akt/eNOS activation was rescued by GDF6 overexpression (Figure 5G). Conversely, augmented VEGFR2/Akt/eNOS phosphorylations were observed in HUVECs treated with the supernatants of *SCDAL* overexpressing hES‐MSCs, and this was neutralized by GDF6 knockdown in *SCDAL* overexpressing hES‐MSCs (Figure 5H).

To further explore the role of VEGFR2 in the GDF6‐mediated angiogenic activity, we used ZM323881, a selective inhibitor of VEGFR2 tyrosine kinase, that can potently block activation of Akt and eNOS.^[^ ^23^ ^]^ To determine the optimal concentration of ZM323881, HUVECs were pretreated with escalating doses of ZM323881 for 1 h, followed by tube formation assays under basal condition in the presence of ZM323881. Compared with DMSO control, a dose of 1 µm ZM323881 did not inhibit endothelial tube formation under basal condition, but blocked VEGFR2/Akt/eNOS activation induced by hES‐MSC supernatants (Figure S9F,G, Supporting Information), and markedly abolished the angiogenic effects of hES‐MSC supernatants on HUVECs (Figure 5I,J; Figure S10A–F, Supporting Information).

These results indicated that *SCDAL*‐mediated GDF6 secretion from hES‐MSCs promoted endothelial angiogenesis via VEGFR2 dependent signaling.

### 2.7 *SCDAL* Regulates GDF6 Expression by Recruiting SNF5 to the GDF6 Promoter

**Figure 6:**
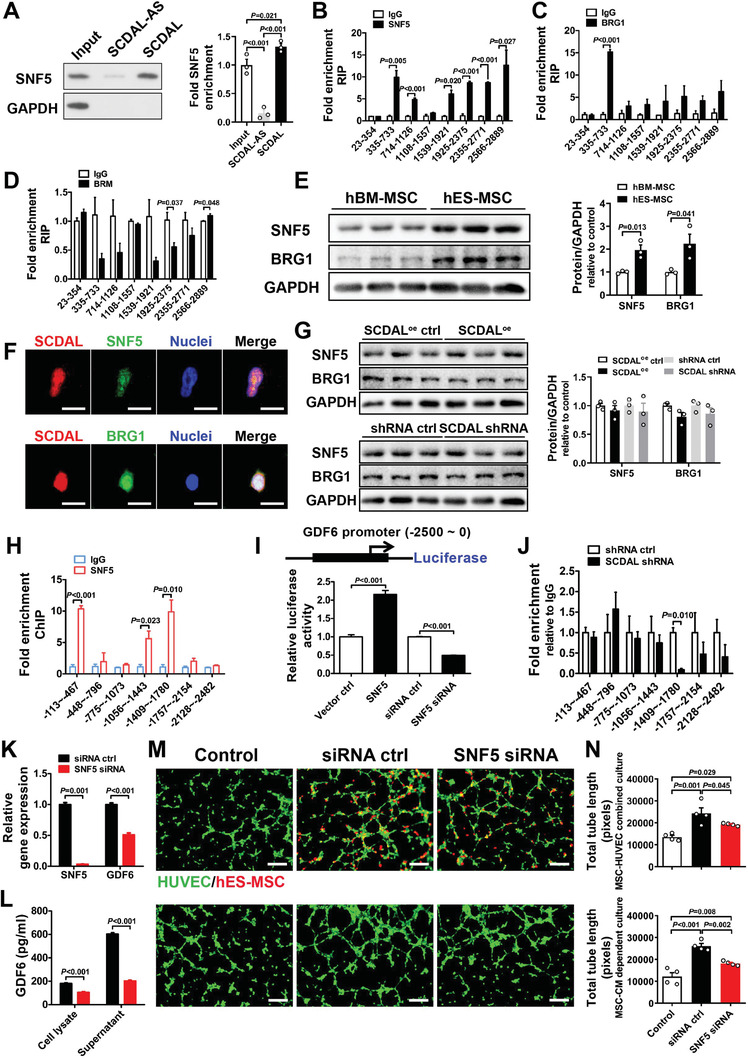
*SCDAL* associates with SNF5 to initiate GDF6 expression. A) Quantification of SNF5 in the pulled‐down protein fraction after western blot analysis (*n* = 3). B–D) RIP verification of the physical interaction between *SCDAL* and SNF5, BRG1, or BRM in hES‐MSC lysates (*n* = 3). E) Comparison of SNF5 and BRG1 protein levels between hBM‐MSCs and hES‐MSCs (*n* = 3). F) Respective colocalization of *SCDAL* (red) with SNF5 (green) or BRG1 (green) in hES‐MSCs. Scale bar, 10 µm. G) SNF5 and BRG1 expression in hES‐MSCs after *SCDAL* overexpression or knockdown (*n* = 3). H) ChIP‐qPCR examination of SNF5 occupation at the GDF6 promoter in hES‐MSCs (*n* = 3). I) The GDF6 promoter is constructed into a luciferase reporter and subjected to luciferase assays in hES‐MSCs after SNF5 overexpressing (SNF5) or knockdown (SNF5 siRNA) (*n* = 4). J) ChIP‐qPCR confirmation of SNF5‐binding regions on the GDF6 promoter in *SCDAL* shRNA hES‐MSCs (*n* = 3). K,L) qRT‐PCR and ELISA examination of GDF6 expression and secretion in hES‐MSCs after SNF5 knockdown (*n* ≥ 3). M,N) Representative images and quantification for total HUVEC (green) tube length after cocultured with SNF5 depleted hES‐MSCs (red, top panels) or their conditioned supernatants (bottom panels) as compared to control groups (*n* = 4). Scale bar, 200 µm. All bars in (A–E), (G–L), and (N) represent mean ± SEM ((B–E) and (H–L), unpaired Student's *t*‐test; (A), (G), and (N), One‐way ANOVA, LSD, S‐N‐K, and Waller–Duncan analysis).

To establish the molecular mechanisms for *SCDAL*‐dependent GDF6 regulation, we performed RNA pull‐down assays with biotinylated *SCDAL* followed by mass spectrometry. Using *SCDAL* antisense as a control, we detected 273 proteins specifically interacted with *SCDAL* in hES‐MSC lysates (Figure S11A, Supporting Information). Given that *SCDAL* might play a role in transcriptional regulation deduced from its nuclear localization, we focused on transcriptional factors, DNA‐binding proteins, and chromatin‐associated proteins in these *SCDAL* interacting proteins. Among 17 selected proteins, SNF5 (also known as SMARCB1/BAF47/INI1), a core subunit of SWItch/sucrose nonfermentable (SWI/SNF) adenosine triphosphate (ATP)‐dependent chromatin‐remodeling complexes,^[^ ^24^ ^]^ aroused our interests because the catalytic subunit of SWI/SNF complexes BRG1 participated in lncRNA‐mediated endothelial angiogenic function.^[^ ^7a^ ^]^ We speculated that SNF5 might be involved in *SCDAL*‐mediated angiogenesis. SNF5 was found to strongly interact with *SCDAL* in the pulled‐down protein fraction by western blot (**Figure** **6**A). We further used RNA immunoprecipitation (RIP) with SNF5 antibody to verify that specific regions of *SCDAL* (335 to 733, 714 to 1126, 1539 to 1921, 1925 to 2375, 2355 to 2771, and 2566 to 2889) were significantly enriched upon SNF5 immuoprecipitation (Figure 6B), suggesting that segment #1 (335 to 1126) and segment #2 (1539 to 2889) of *SCDAL* interacted with SNF5. Furthermore, we investigated the effects of truncated *SCDAL* (1833 to 2916, mainly located within segment #2 of *SCDAL*) on GDF6 expression and angiogenic capacity in hES‐MSCs, and found that overexpression of truncated *SCDAL* in hES‐MSCs could augment GDF6 expression and enhance their angiogenic potential on HUVECs (Figure S11B–D, Supporting Information). However, the effects of truncated *SCDAL* were not as strong as those of full‐length *SCDAL*, indicating that both segment #1 and segment #2 were required for the function of *SCDAL* in hES‐MSCs. Meanwhile, the two binding fragments were predicted to harbor stable stem‐loop structures by RNA folding analysis (Figure S11E, Supporting Information).

Because the SWI/SNF complexes use ATPase subunit BRG1 or BRM to provide ATP for remodeling chromatin and regulating gene transcription, we next investigated whether *SCDAL* interacted with BRG1 or BRM by RIP in hES‐MSC lysates, and found that anti‐BRG1 antibody but not anti‐BRM antibody could precipitate *SCDAL* (Figure 6C,D). SNF5 and BRG1 were also more highly expressed in hES‐MSCs than in hBM‐MSCs (Figure 6E), and colocalized with *SCDAL* in the nuclei of hES‐MSCs (Figure 6F). However, *SCDAL* depletion or overexpression did not alter the expression levels of SNF5 and BRG1, indicating that *SCDAL* was not involved in the post‐translational regulation of SNF5 and BRG1 (Figure 6G). These findings indicated that *SCDAL* was physically associated with a SNF5‐BRG1 complex.

Growing evidence indicates that the SWI/SNF complexes regulate gene transcription by binding to promoter loci and refolding chromatin.^[^ ^24^ ^]^ To investigate the role of SNF5 or BRG1 in *SCDAL*‐induced GDF6 expression, we measured occupation of SNF5 or BRG1 at the GDF6 promoter using ChIP in hES‐MSC lysates, and found specific regions of GDF6 promoter (−113 to −467, −1056 to −1443, and −1409 to −1780 bp) were enriched upon SNF5 immuoprecipitation (Figure 6H). However, there was less evidence for BRG1 binding to the GDF6 promoter (Figure S11F, Supporting Information). Next, we inserted the GDF6 promoter (−2500 to 0 bp) upstream of a luciferase reporter, and found that SNF5 overexpression activated the luciferase activity of GDF6 promoter reporter in hES‐MSCs, whereas SNF5 depletion inhibited the luciferase activity (Figure 6I). Given the high *SCDAL* expression in hES‐MSCs, we speculated that SNF5 depletion and overexpression regulated the luciferase activity driven by GDF6 promoter in the presence of high level of *SCDAL*. Then, we performed ChIP assays with SNF5 antibody in the *SCDAL*‐silenced hES‐MSCs, and found decreased SNF5 occupancy at the −1409 to −1780 loci of GDF6 promoter upon *SCDAL* knockdown (Figure 6J), indicating an essential role of *SCDAL* in recruiting SNF5 to the GDF6 promoter.

Finally, we investigated the biological function of SNF5 in regulating hES‐MSC angiogenesis. Consequently, siRNA‐mediated SNF5 depletion remarkably decreased GDF6 mRNA, as well as intra‐ and extracellular GDF6 protein levels (Figure 6K,L; Figure S11G, Supporting Information). Importantly, SNF5 knockdown did not affect cell viability and proliferation of hES‐MSCs (Figure S11H,I, Supporting Information), but significantly impaired their angiogenic potential on HUVEC tube formation (Figure 6M,N; Figure S11J–M, Supporting Information).

Taken together, our data showed that *SCDAL* initiated GDF6 expression through recruitment of SNF5 to the promoter locus of GDF6 gene, thereby promoting hES‐MSC angiogenesis.

### 2.8 The Biological Impact of *SCDAL*‐Mediated Angiogenesis in Ischemic Hearts

**Figure 7:**
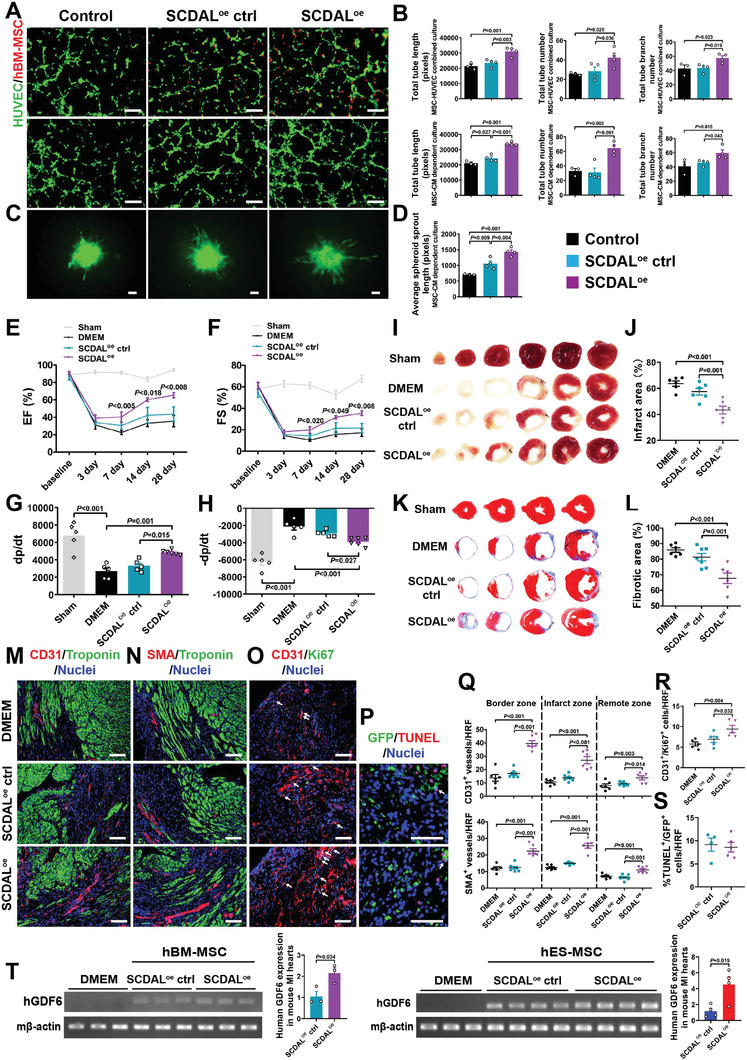
The biological function of *SCDAL* in mediating the angiogenic potential of hBM‐MSCs. A,B) Representative images and quantification for total tube length, tube number, and tube branch number of HUVEC tube formation (green) cocultured with *SCDAL* ^oe^ hBM‐MSCs (red, top panels) or their supernatants (middle panels) as compared to controls (*n* ≥ 3). Scale bar, 200 µm. C,D) Representative images and quantification for average HUVEC spheroid sprout length after cocultured with *SCDAL* ^oe^ hBM‐MSC‐conditioned supernatants as compared to control groups (*n* ≥ 3). Scale bar, 200 µm. E–H) Echocardiographical and hemodynamic assessments of EF, FS, and ±dp/dt in Sham (*n* ≥ 5), DMEM (*n* = 5), *SCDAL* ^oe^ ctrl hBM‐MSC (*n* = 5), and *SCDAL* ^oe^ hBM‐MSC (*n* = 6)‐receiving mice. I–L) TTC staining and Masson's trichrome staining on serial heart sections four weeks post MI with corresponding quantifications in Sham, DMEM (*n* = 6), *SCDAL* ^oe^ ctrl hBM‐MSC (*n* ≥ 6), and *SCDAL* ^oe^ hBM‐MSC (*n* = 6)‐receiving mice. M,N,Q) Representative border zone images and quantification for CD31^+^ and SMA^+^ vessels in DMEM (*n* = 6), *SCDAL* ^oe^ ctrl hBM‐MSC (*n* = 7), and *SCDAL* ^oe^ hBM‐MSC (*n* = 6)‐receiving mice 28 days after MI. Scale bar, 100 µm. O,R) Representative images and quantification for CD31^+^/Ki67^+^ cells (white arrows) in DMEM (*n* = 5), *SCDAL* ^oe^ ctrl hBM‐MSC (*n* = 5), and *SCDAL* ^oe^ hBM‐MSC (*n* = 5)‐treated hearts at 3 days post‐MI. Scale bar, 100 µm. P,S) Representative images and quantification for TUNEL^+^/GFP^+^ cells (white arrows) in different hBM‐MSC (*n* ≥ 4)‐treated hearts three days after transplantation. Scale bar, 100 µm. HRF, high resolution field. T) Semi‐quantitative RT‐PCR and qRT‐PCR detection of human GDF6 expression in DMEM (*n* = 3), *SCDAL* ^oe^ ctrl hBM‐MSC/hES‐MSC (*n* ≥ 3), and *SCDAL* ^oe^ hBM‐MSC/hES‐MSC (*n* ≥ 3)‐treated hearts at 3 days post infarction. Mouse *β*‐actin is used as a loading control. All bars in (B), (D–H), (J), (L), and (Q–T) represent mean ± SEM ((B), (D–H), (J), (L), and (Q–R), One‐way ANOVA, LSD, S‐N‐K, and Waller–Duncan analysis; (S) and(T), unpaired Student's *t*‐test).

To demonstrate the biological significance and therapeutic potential of *SCDAL* function in angiogenesis, we overexpressed *SCDAL* in hBM‐MSCs ( ≈60‐fold increase; Figure S12A, Supporting Information), and found that *SCDAL* ^oe^ did not affect cell viability and proliferation of hBM‐MSCs (Figure S12B,C, Supporting Information), but enhanced their effects on HUVEC angiogenesis (**Figure** **7**A,B) and spheroid sprouting (Figure 7C,D). Following transplantation, infarcted mice treated with *SCDAL* ^oe^ hBM‐MSCs showed robust improvements in cardiac function (EF, FS, and ±dp/dt; Figure 7E–H; Figure S12D, Supporting Information), infarct area and fibrotic area (Figure 7I–L), and neovascularization (Figure 7M,N,Q; Figure S12E, Supporting Information) four weeks after MI as well as endothelial proliferation three days after MI (Figure 7O,R) compared to control hBM‐MSC and vehicle‐receiving mice. Also, *SCDAL* ^oe^ did not affect the survival of transplanted hBM‐MSCs in the infarcted myocardium (Figure 7P,S). Moreover, elevated GDF6 expression and secretion was detected in *SCDAL* ^oe^ hBM‐MSCs compared to control hBM‐MSCs (Figure S12F,G, Supporting Information).

Although gene manipulation of *SCDAL* in hES‐MSCs/hBM‐MSCs did not affect their survival three days after transplantation, we demonstrated enhanced GDF6 expression in *SCDAL* ^oe^ hBM‐MSC/hES‐MSC‐treated mouse hearts (Figure 7T), further indicating an important role of *SCDAL*‐mediated GDF6 paracrine signaling in promoting angiogenesis.

**Figure 8:**
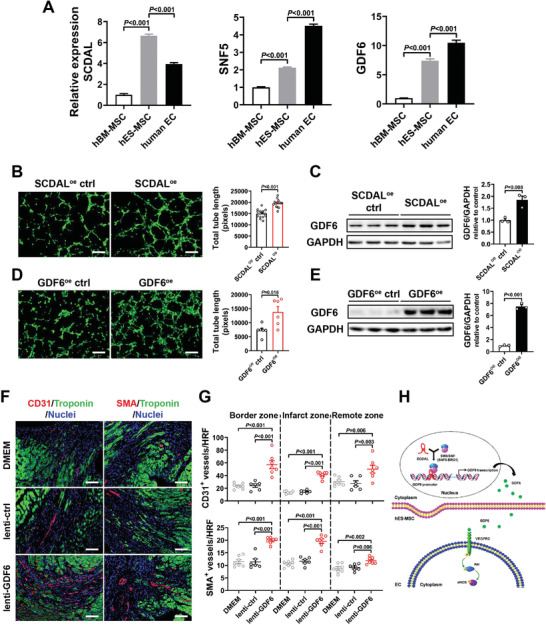
*SCDAL*‐GDF6 directly enhances endothelial angiogenesis. A) The expression levels of *SCDAL*, SNF5, and GDF6 in different cells (*n* = 3). B,D) Representative images and quantification for HUVEC tube formation after *SCDAL* (*n* = 15) or GDF6 (*n* = 6) overexpression. C,E) Elevated GDF6 protein levels in *SCDAL* ^oe^ or GDF6^oe^ HUVECs (*n* = 3). F,G) Representative border zone images and quantification for vascular densities in mouse hearts treated with DMEM (*n* = 7), lenti‐ctrl (*n* = 6), and lenti‐GDF6 (*n* = 7) at 28 days after MI. Scale bar, 100 µm. H) Schematic model of *SCDAL*‐regulated GDF6 function in mediating angiogenesis. In hES‐MSC nucleus, *SCDAL* facilitates the recruitment of SNF5 to the GDF6 promoter, thereby initiating GDF6 transcription. The secreted GDF6 of hES‐MSCs promotes endothelial angiogenesis through VEGFR2/Akt/eNOS pathway. All bars in (A–E) and (G) represent mean ± SEM ((A) and (G), One‐way ANOVA, LSD, S‐N‐K, and Waller–Duncan analysis; (B–E), unpaired Student's *t*‐test).

Next, given the different expression patterns of *SCDAL*, SNF5, and GDF6 in human endothelial cells relative to hES‐MSCs (**Figure** **8**A), we investigated whether *SCDAL‐*GDF6 directly affected endothelial angiogenesis. We respectively overexpressed *SCDAL* or GDF6 in HUVECs (Figure 8E; Figure S13A, Supporting Information), and found both gene manipulations did not affect their viability and proliferation (Figure S13B,C, Supporting Information), but enhanced tube formation (Figure 8B,D) and elevated GDF6 protein levels after *SCDAL* overexpression (Figure 8C) in HUVECs. Furthermore, intramyocardial injection of lentiviruses encoding GDF6 significantly increased CD31^+^ and SMA^+^ vessel densities in the border, infarct, and remote areas of the infarcted hearts four weeks post infarction (Figure 8F,G; Figure S13D,E, Supporting Information).

These findings strongly supported the biological impact and therapeutic potential of *SCDAL*‐GDF6 axis in promoting angiogenesis.

## 3 Discussion

Although hES‐MSCs and hBM‐MSCs are largely indistinguishable in all essential characteristics (e.g., marker and HLA expression, multipotency), hES‐MSCs are generally more potent for the treatment of ischemic cardiac injury. The results presented here provide the first lncRNA focused molecular characterization of MSCs from different origins, and reveal for the first time that the enhanced angiogenic potency of hES‐MSCs is at least partially attributable to higher levels of *SCDAL* expression. We further demonstrate that *SCDAL* recruits the SWI/SNF chromatin‐remodeling protein SNF5 to the GDF6 promoter in hES‐MSCs, and up‐regulates GDF6 expression and secretion. The secreted GDF6 promotes the angiogenic activity of endothelial cells via VEGFR2/Akt/eNOS activation (Figure 8H). Furthermore, the angiogenic potency of hBM‐MSCs and HUVECs can be improved by *SCDAL* overexpression associated with elevated GDF6 induction, and GDF6 overexpression can enhance endothelial angiogenesis in vitro and in vivo, indicating the biological prevalence of *SCDAL*‐GDF6 function in mediating angiogenesis. Collectively, these observations point out a previously unknown lncRNA‐mediated paracrine signaling involving the *SCDAL*‐GDF6 axis.

The importance of lncRNAs as regulators in angiogenesis and vascular diseases is increasingly recognized.^[^ ^6^, ^7^, ^8^, ^9^ ^]^ These angiogenic lncRNAs have very diverse functions depending on their subcellular localization. For example, as a more nucleus‐enriched lncRNA, *LncEGFL7OS* regulates human endothelial angiogenesis by interaction with MAX transcription factor at the EGFL7/miR‐126 promoter locus and enhancing their transcription.^[^ ^7c^ ^]^ Another nuclear‐expressed lncRNA *SNHG12* has been identified as a homeostatic regulator of genomic stability in atherosclerotic lesions by interaction with DNA‐dependent protein kinase, a key mediator of the DNA damage response.^[^ ^7d^ ^]^ In our study, we identify SNF5 as a *SCDAL*‐associated protein. SNF5 (SMARCB1) is required for the integrity and function of SWI/SNF complexes and is present in all known variants of the complex, and is essential for SWI/SNF‐mediated promoter/enhancer activation.^[^ ^25^ ^]^ Down‐regulation of *SCDAL* in hES‐MSCs decreases SNF5/GDF6 promoter interaction at the −1409 to −1780 loci and GDF6 expression, suggesting that *SCDAL*‐SNF5 interaction is critical for transcriptional regulation of GDF6. Recently, two potential motifs have been defined by MEME‐ChIP motif analysis in SMARCB1 ChIP‐seq experiment in human BJ fibroblasts.^[^ ^26^ ^]^ However, the −1409 to −1780 loci of GDF6 promoter does not contain these motifs. Considering that the cell type and biological process are different from that study, detailed information of SNF5‐binding motif(s) on GDF6 promoter needs further investigation. Furthermore, the present results are not sufficient to answer the questions whether *SCDAL* facilitates SNF5‐BRG1 complex formation and whether the interaction between *SCDAL* and BRG1 is important for the function of SNF5 in promoting GDF6 expression. Based on a recent study suggesting the possibility of a general mechanism in which SWI/SNF complexes cooperate with lncRNA to achieve transcriptional activation,^[^ ^26^ ^]^ these questions will be great research points for investigators in future studies.

LncRNAs have been proposed to act as scaffolds that coordinate distinct chromatin‐modifying complexes to target DNA loci.^[^ ^27^ ^]^ For example, *lncBRM* interacted with BRM (the ATPase subunit of SWI/SNF complex) to activate YAP1 signaling and promote self‐renewal of liver cancer stem cells, and the binding fragment was predicted to form stable stem‐loop structure.^[^ ^28^ ^]^ The binding fragments of *SCDAL* and SNF5 are predicted to harbor stable stem‐loop structures which may facilitate the binding of SNF5 to the promoter of GDF6.

GDF6 is a member of the transforming growth factor *β* superfamily. Mutations in the GDF6 gene are associated with vascular aberrant development,^[^ ^29^ ^]^ which have been proposed as a cause of age‐related macular degeneration and ocular developmental anomalies.^[^ ^30^ ^]^ GDF6 expression declines in the MSCs of aging mice. GDF6 up‐regulation in MSCs from older animals improves their differentiation potential, and intraperitoneal injection of lentiviruses coding for hGDF6 in geriatric mice leads to cerebrovascular improvements and attenuates the severity of a number of age‐related pathological conditions.^[^ ^31^ ^]^ GDF6 secreted by NADPH oxidase‐2‐activated fibroblasts can induce vascular smooth muscle cell growth and remodeling.^[^ ^32^ ^]^ GDF6 may also promote endothelial vascular integrity, because both total and Tyr1175‐phosphorylated VEGFR2 accumulate in GDF6‐deficient endothelial cells, which subsequently promote ERK phosphorylation and endothelial instability.^[^ ^33^ ^]^ Here we report for the first time that *SCDAL*‐mediated GDF6 secretion from hES‐MSCs functions as a novel ligand for VEGFR2 and confers rapid VEGFR2 phosphorylation at Tyr951 rather than Tyr1175, representing non‐canonical VEGFR2 activation,^[^ ^20^ ^]^ and subsequently induces the downstream Akt/eNOS cascade, thereby promoting endothelial cells to initiate angiogenesis. Intramyocardial injection of GDF6 overexpressing lentiviruses significantly enhanced myocardial angio‐ and arteriogenesis in the infarcted mouse hearts. These findings add to our current knowledge of GDF6‐mediated endothelial regulation in cardiovascular system, and the potential binding sites between GDF6 and VEGFR2 will provide clues for further exploring the mechanism of GDF6‐driven VEGFR2 activation.

Emerging studies have demonstrated that lncRNAs can be transcriptionally regulated by key transcriptional factors such as p53, nuclear factor kappaB, Sox2, Oct4, and Nanog.^[^ ^34^ ^]^ From the UCSC and PROMO databases, we have found several putative transcriptional factor‐binding sites within the *SCDAL* promoter region, such as E2F1 (data not shown), a typical transcriptional factor for several reported lncRNAs.^[^ ^35^ ^]^ Elucidating the transcriptional mechanisms of *SCDAL* would allow further investigation of how transcriptional factors (e.g., E2F1) and *SCDAL* coordinate to regulate the angiogenic capacity in hES‐MSCs.

It is interesting to note that different numbers of genes were affected by *SCDAL* knockdown versus overexpression in hES‐MSCs. This differential effect may indicate the heterogeneity in sensitivity to *SCDAL* expression among its downstream target genes. Exploring the full extent of *SCDAL* effect in downstream gene regulation and identifying the genome‐wide targets of *SCDAL* (e.g., through ChIRP‐seq) will be an important aim for future studies. Although our study has focused on MSCs expressed lncRNAs associated with angiogenesis, protein‐coding mRNAs will also have significant contributions. It is likely that interactions between lncRNA and mRNA products are responsible for differential impact on angiogenic remodeling, and will need to be explored in future studies.

## 4 Conclusion

In summary, we demonstrate an important role for *SCDAL*‐GDF6 in mediating enhanced angiogenesis. The insights obtained from this study advance our understanding of the physiological roles of lncRNAs in general and the growing importance of these molecules in angiogenesis.

## 5 Experimental Section

A detailed description of materials and methods is provided in the Supporting Information.

### Study Approval

Experiments involving live animals were performed in accordance with the Guide for the Care and Use of Laboratory Animals published by the US National Institutes of Health (NIH Publication No.85‐23, revised 1996), and were approved by the Institutional Animal Care and Use Committee of Second Affiliated Hospital of Zhejiang University (No. 2017‐340). Experiments involving hBM‐MSCs were preformed with informed consent according to the guidelines approved by the Ethics Committee of Second Affiliated Hospital, College of Medicine, Zhejiang University (No. 2015‐011).

### Statistical Analysis

All results were presented as mean value ± standard error of the mean (SEM) using GraphPad Prism. Comparisons between two experimental groups were evaluated for significance via the unpaired Student's *t*‐test, whereas comparisons among three or more groups were evaluated for significance via one‐way analysis of variance (ANOVA) followed by LSD, S‐N‐K, and Waller–Duncan analysis with SPSS softwares. A value of *p* < 0.05 was considered statistically significant. The sample size was ≥4 for in vivo animal studies, and ≥3 for in vitro studies.

## Conflict of Interest

The authors declare no conflict of interest.

## Supporting information

Supporting Information

## Data Availability

Research data are not shared.
