# Supplementary material for: A Novel Human Long Noncoding RNA SCDAL Promotes Angiogenesis through SNF5‐Mediated GDF6 Expression
Source: Adv Sci (Weinh). 2021 Jul 28;8(18):2004629. doi: 10.1002/advs.202004629 (PMC8456203; doi:10.1002/advs.202004629)
Supplement: Supplementary file 1 — Supporting Information [file ADVS-8-2004629-s001.pdf]

© 2021 Wiley-VCH GmbH

ADVANCED SCIENCE
Open Access

Supporting Information

for *Adv. Sci.,* DOI: 10.1002/advs.202004629

A Novel Human Long Noncoding RNA *SCDAL* Promotes Angiogenesis Through SNF5-mediated GDF6 Expression

Rongrong Wu, Wangxing Hu, Huan Chen, Yingchao Wang, Qingju Li, Changchen Xiao, Lin Fan, Zhiwei Zhong, Xiaoying Chen, Kaiqi Lv, Shuhan Zhong, Yanna Shi, Jinghai Chen, Wei Zhu, Jianyi Zhang, Xinyang Hu,* and *Jian'an Wang**

Supporting Information

A Novel Human Long Noncoding RNA *SCDAL* Promotes Angiogenesis Through SNF5-mediated GDF6 Expression

Rongrong Wu, Wangxing Hu, Huan Chen, Yingchao Wang, Qingju Li, Changchen Xiao, Lin Fan, Zhiwei Zhong, Xiaoying Chen, Kaiqi Lv, Shuhan Zhong, Yanna Shi, Jinghai Chen, Wei Zhu, Jianyi Zhang, Xinyang Hu, Jian'an Wang**

Materials and Methods

Derivation and characterization of hES-MSCs

Two NIH-registered hESC lines, H9 and H14 (generously provided by Professor Lei Xiao from Zhejiang University), were maintained and expanded on Matrigel-coated plates in feeder-free mTeSR1 medium (STEMCELL Technologies). For the derivation of hES-MSCs, a Rho-associated protein kinase inhibitor Y-27632-assisted monolayer culture system[1] with modifications was adopted. Briefly, hESCs were treated with 10 μM Y-27632 (Sigma-Aldrich) for 1 hour, dissociated into single cells with ACCUTASE™ cell detachment solution (STEMCELL Technologies), seeded at 2-3×10^4 cells/cm^2 onto 0.1% gelatin (Sigma-Aldrich)-coated dishes in hESC medium containing 10 μM Y-27632 for another 12 hours, and then transferred to high glucose Dulbecco's Modified Eagle Medium (DMEM, Thermo Fisher Scientific, Invitrogen) supplemented with 10% fetal bovine serum (FBS, Thermo Fisher Scientific, Invitrogen) for continue culture. The differentiated cells were maintained with medium exchange every other day until becoming spindle-shape morphology, and passaged at 80-90% confluence in a 1:2-1:3 ratio. Characterization experiments were performed after 3-5 passages of culture.

For flow cytometry analysis, hES-MSCs were suspended as 5×10^5 cells/100 μl in phosphate-buffered saline (PBS) containing 1% FBS. Cells were separately labeled with human fluorochrome-conjugated primary antibodies (Table S2), including CD29-phycoerythrin (PE), CD34-PE, CD44-PE, CD45-fluorescein isothiocyanate (FITC), CD90-allophycocyanin (APC), CD105-PE, CD117-PE, CD133-APC, CD166-PE, HLA-ABC-FITC and HLA-DR-PE at 4 ^{o}C for 45 minutes. After that, cells were washed twice in PBS and at least 10,000 events were acquired for each sample using LSRII Flow Cytometry (BD Biosciences). Conjugated isotype antibodies were used as negative controls. Data were analyzed by FlowJo software (Tree Star, Inc.).

The multipotency of hES-MSCs was evaluated via standard published protocols with minor modifications[1]. Osteogenesis was induced in monolayer hES-MSCs in DMEM containing 10% FBS, 100 nM dexamethasone, 50 μM ascorbic acid and 10 mM β-glycerophosphate (Sigma-Aldrich). Adipogenesis was achieved by treating hES-MSCs with DMEM containing 10% FBS, 1 μM dexamethasone, 0.2 mM indomethacin and 10 μg/ml insulin (Sigma-Aldrich). Chondrogenesis was induced by culturing hES-MSCs in DMEM containing 10% FBS, 10 ng/ml recombinant human transforming growth factor-β3 (PeproTech), 100 nM dexamethasone and 50 μM ascorbic acid. At 14-21 days, osteogenic, adipogenic and chondrogenic cultures were fixed and respectively stained in Alizarin Red (cyagen), Oil Red O (Sigma-Aldrich) and Toluidine Blue solutions.

Culture of hBM-MSCs and HUVECs

hBM-MSCs were isolated from bone marrow aspirates that were obtained from caput femoris upon hip replacement surgery with informed consent according to the guidelines approved by the Ethics Committee of Second Affiliated Hospital, College of Medicine, Zhejiang University (No. 2015-011) as described previously[2]. Briefly, whole bone marrow aspirates were washed with PBS, centrifuged at 1000 rpm for 5 minutes, and plated in 75 cm^2 flasks in 10% FBS-supplemented DMEM. After 2 days, the nonadherent cells were removed and the adherent cells were maintained at 37°C, 5% CO_2 with medium change every three days until colony confluence, and passaged in a 1:2-1:3 ratio. Characterizations of surface marker expression and multipotency were performed after 3-5 passages of culture as described above. Donor characteristics and passages of hBM-MSCs were listed in Table S1.

Human ECs and HUVECs were maintained in low glucose DMEM (Thermo Fisher Scientific, Invitrogen) containing 10% FBS. HUVECs were transfected with lentivirus encoding green fluorescent protein (GFP) expression (GENECHEM Corp, China) for subsequent tube formation assay.

lncRNA sequencing and data analysis

Total RNA of hES-MSCs from three differentiation experiments of H14 hESCs and hBM-MSCs from three different donors were extracted. First, ribosomal RNA (rRNA) was removed by Ribo-Zero™ rRNA Removal Kit (Epicentre). Sequencing libraries were prepared from the rRNA-depleted RNA by NEBNext® Ultra™ Directional RNA

Library Prep Kit for Illumina® (New England Biolabs) following manufacturer's recommendations, and then sequenced on the Illumina Hiseq2000 platform by Novogene (China) and 100 bp paired-end reads were generated. Clean data (clean reads) were obtained through in-house perl scripts by removing reads containing adapter, reads containing ploy-N and low quality reads from raw data, and aligned to the *homo sapiens* GRCh38.p12 genome using TopHat2 v2.0.9[3]. The mapped reads of each sample were assembled by both Scripture (beta2)[4] and Cufflinks (v2.1.1)[5]. Transcripts predicted with coding potential by either/all of the four tools [Coding-Non-Coding-Index (CNCI) (v2)[6], CPC (0.9-r2)[7], Pfam Scan (v1.3)[8] and PhyloCSF (v20121028)[9]] were filtered out, and those without coding potential were candidate set of lncRNAs. Differential expression analysis was performed by Cuffdiff (v2.1.1) between hES-MSCs and hBM-MSCs, and transcripts of lncRNAs with fold change >2 and an adjusted *P* value <0.05 were assigned as differentially expressed.

In vitro Matrigel tube formation assay

Tube formation was evaluated in HUVECs cocultured with MSCs (***Protocol 1***) or with the conditioned media (CM) from MSCs (***Protocol 2***). ***Protocol 1*****:** indicated hES-MSCs or hBM-MSCs were labeled with a PKH26 Red Fluorescent Cell Linker Kit (Sigma-Aldrich) as directed by the manufacturer's instructions. $0.7\text{-}0.8\times10^4$ GFP-expressing HUVECs and PKH26-labeled hES-MSCs/hBM-MSCs mixture at 3: 1 ratio were plated onto per well of μ-Slide Angiogenesis (ibidi GmbH) pre-coated with growth factor-reduced Matrigel™ (Corning Incorporated). ***Protocol 2*****:** indicated

hES-MSC/hBM-MSC-derived CM were collected in DMEM containing 1% FBS for 48 hours, and precleared of cellular debris and particulate matter by centrifugation at 2000 rpm for 10 minutes. 0.7-0.8×10^4 GFP-expressing HUVECs per well were seeded with the addition of indicated hES-MSC/hBM-MSC-derived CM, or DMEM containing 1% FBS as negative control. All plates were incubated at 37 ^{o}C in a humidified incubator supplied with 5% CO_2 for 6-8 hours. Total tube length, tube number and tube branch number of capillary-like structures under high resolution field (HRF) per well were quantitatively measured by Image-Pro Plus software 6.0. At least three wells were used for each condition and results are representative of the mean of each at least three well group. The experiments were repeated for at least three times with similar results.

Spheroid-based spouting assay

HUVEC spheroids were generated as described previously[10]. Briefly, HUVECs were cultured as hanging drops (400 cells/25 μl per drop) in culture medium containing 0.24% (w/v) methylcellulose for 24 hours to form spheroids. The spheroids were collected and re-suspended in methocel containing 20% FBS. A collagen stock solution was prepared by mixing 8 volume of collagen extract of rat tails (3.83 mg/ml, Corning Incorporated) with 1 volume of 10× M199 (Sigma-Aldrich) on ice, and ~1 volume of 0.2 N NaOH to adjust the pH to 7.4, after which it was mixed with equal volume of spheroid-containing methocel solution. The spheroid-containing collagen was rapidly transferred into 24-well plates (1 ml per well) and allowed to polymerize

(4 hours), after which 0.1 ml indicated hES-MSC/hBM-MSC-derived CM or DMEM containing 1% FBS was added on top of each gel. The gels were incubated at 37 ^{o}C, 5% CO_2, and 100% humidity for 24 hours. For quantification of the length of sprouts, six to eight randomly selected spheroids per well were acquired under a fluorescence microscope and analyzed as average spheroid sprout length per well with Image-Pro Plus software 6.0.

RNA extraction and qRT-PCR

Total RNA was extracted from cells using TRIzol reagent (Thermo Fisher Scientific, Invitrogen). 2 μg of total RNA was reverse-transcribed to cDNA with M-MLV Reverse Transcriptase (Takara). Resultant cDNA samples were subjected to qPCR on 7500 Fast Real-Time PCR System (Applied Biosystems) using SYBR® Premix Ex Taq™ (Takara) and the primers listed in Table S3. β-actin levels were measured to serve as an internal control, and the relative expression levels were calculated using the $2^{-\Delta\Delta Ct}$ method.

Western blot

Cells were lysed in RIPA lysis buffer supplemented with Pierce™ protease inhibitors (Thermo Fisher Scientific) and, after centrifugation, the supernatants were collected and quantified using the BCA protein assay. 20-30 μg of protein per sample was separated via sodium dodecyl sulfate-polyacrylamide gel electrophoresis, transferred to a poly-vinylidene fluoride membrane, and blocked with 5% milk for 1 hour. The

membranes were incubated with indicated primary antibodies (Table S2) overnight at 4 ^{o}C, and blotted with the corresponding horseradish peroxidase-conjugated secondary antibodies at room temperature for 2 hours. Afterwards, the protein bands could be visualized via enhanced chemiluminescence.

RACE

Total RNA was extracted from hES-MSCs, and converted into first strand cDNA with an M-MuLV First Strand cDNA Synthesis Kit (Sangon Biotech, China). Nested 5' and 3' RACE products were obtained using LA Taq® polymerase with GC Buffer (Takara) and the primers listed in Table S4. The gel products were extracted with a Gel Extraction kit (Sangon Biotech), cloned into pMD18-T vectors and verified by Sanger sequencing.

Northern blot

Total RNA was extracted from hBM-MSCs and hES-MSCs using standard TRIzol methods. *SCDAL* probe was generated by RT-PCR using the following primers: forward 5'-GGATGTAATCACGCACCCCATG-3' and reverse 5'-ACGATGGCACCCACTGATGTCT-3', then denatured and labeled with digoxigenin (DIG) using a DIG High Primer DNA Labeling and Detection Starter Kit II (Sigma-Aldrich, Roche). For northern blot, samples were separated by electrophoresis with formaldehyde denaturing agarose gel, and transferred to a

positively charged nylon membrane (Merck Millipore) with 10× saline-sodium citrate (SSC) buffer. After crosslinked by ultraviolet, membrane was incubated with hybrid buffer for 4 hours of prehybridization, followed by incubation with DIG-labeled probes overnight at 42 ^{o}C. The detection was performed using the above kit according to the manufacturer's instructions.

Detecting potentially translated ORFs of *SCDAL*

ORFs in the full-length *SCDAL* were predicated by NCBI ORFfinder (https://www.ncbi.nlm.nih.gov/orffinder/) with standard setting. Three most potentially translated ORF sequences (ORF1: 543 to 839 bp, ORF2: 2072 to 2320 bp, and ORF3: 784 to 990 bp) were respectively cloned into a GV219 vector and constructed with the Flag, 6His and HA tags at the C terminus by GENECHEM Corp. For the detection of *SCDAL*-ORF1-Flag fusion protein, *SCDAL*-ORF2-6His fusion protein, and *SCDAL*-ORF3-HA fusion protein, the *SCDAL*-ORF1-Flag, *SCDAL*-ORF2-6His, and *SCDAL*-ORF3-HA constructs were respectively transfected into HEK293T cells for 48 hours, and western blot was performed in whole cell lysates using anti-Flag, -6His and -HA antibodies (Table S2). Truncated protein-coding genes with similar length (NM_032181: 228 bp, NM_003882: 228 bp, and NM_016617: 288 bp) were respectively constructed with the Flag, 6His and HA tags and used as positive controls.

RNA FISH

Cy3-conjugated probes for RNA FISH were designed and synthesized by RiboBio CO., Ltd (China). RNA FISH assay was performed following the Ribo^{TM} lncRNA FISH Probe Mix protocol (RiboBio). Briefly, hES-MSCs were fixed in 10% formaldehyde, permeabilized with ice-cold 0.5% Triton X-100 in PBS, blocked in Pre-hybridization Buffer for 30 minutes at 37 ^{o}C, and incubated in Hybridization Buffer with either *SCDAL* probe or a nuclear (*U6*)/cytoplasmic (*18S*) control probe at 37 ^{o}C in the dark overnight. Then primary and secondary antibodies were added after RNA hybridization for colocalization of *SCDAL* with indicated proteins. Treated samples were counterstained with Hoechst 33258 (Thermo Fisher Scientific, Invitrogen) and visualized by Leica confocal microscopy.

RNA fractionation

Nuclear and cytoplasmic RNA fractions of hES-MSCs were isolated using a PARIS^{TM} Kit (Thermo Fisher Scientific, Invitrogen), according to the manufacturer's instructions. 0.5-1×10^7 freshly cultured cells were collected, lysed thoroughly in ice-cold Cell Fractionation Buffer and centrifuged at 4 ^{o}C and 500 g for 5 minutes. The supernatant was collected as the cytoplasmic fraction, while nuclear pellet was washed and homogenized in ice-cold Cell Disruption Buffer. Both cytoplasmic fraction and nuclear lysate were respectively mixed with an equal volume of 2× Lysis/Binding Solution for further RNA isolation. The *SCDAL* content in the fractions was determined via standard qRT-PCR protocols. β-actin and glyceraldehyde phosphate dehydrogenase (GAPDH) levels were used as cytoplasmic controls,

whereas U6 level was used as a nuclear control.

Lentivirus construction and transfection

Lentiviral vectors expressing either *SCDAL* shRNA, full-length *SCDAL*, GDF6 or VEGFR2 were constructed and generated by GENECHEM Corp. For lentiviral infection, cells were plated as $1\times10^4/cm^2$, infected with indicated lentiviruses or lentiviruses containing empty vectors (control) at multiplicity of infection 30-50 with polybrene for 24 hours, and then refreshed with fresh medium. Transfection efficacy was evaluated after 72 hours of infection via qRT-PCR or western blot.

Synthesis and transfection of Ribo™ lncRNA Smart Silencer and siRNAs

Ribo™ lncRNA Smart Silencer for *SCDAL* was purchased from RiboBio (Table S5). siRNAs targeting GDF6 and SNF5 were designed and synthesized by GenePharma Corp (China, Table S6). Smart Silencer, indicated siRNAs and their scramble controls were transfected into cells at 50 nM final concentration with Lipofectamine® RNAiMAX Reagent (Thermo Fisher Scientific).

CRISPR-on assay

CRISPR/Cas9 Synergistic Activation Mediator (SAM)[11] was used to activate *SCDAL* expression in hES-MSCs. Lentivirus-dCAS9-VP64 and lentivirus-*SCDAL*-single guide RNA (sgRNA) were constructed and generated by GENECHEM Corp. Cells were firstly infected with lentivirus-dCAS9-VP64 and selected with puromycin. The

stable sub-lines were then infected with lentivirus-*SCDAL*-sgRNA to specifically target *SCDAL*. Total RNA was isolated seven days after transfection and subjected to qRT-PCR. The sequence of *SCDAL*-sgRNA: AGCAAGGTGCTAACCCAACA.

Cell viability and proliferation assays

Cell viability of hES-MSCs, hBM-MSCs and HUVECs after genetic modification was determined via a Leica fluorescent microscope by using the LIVE/DEAD® Viability/Cytotoxicity Assay Kit (Molecular Probes, Invitrogen) as directed by the manufacturer's instructions. Indicated cells were stained with the vital dyes calcein acetoxymethylester (calcein AM) and ethidium homodimer-1 (EthD-1) to visualize live (green) and dead (red) cells, respectively. At least >1000 cells were counted from three to five independent replicates for each condition tested.

Indicated transfected hES-MSCs, hBM-MSCs and HUVECs were respectively seeded at a density of 5,000 cells/well in a 96-well plate. At day 0, 1, 3 and 5, 10 μl cell counting kit-8 (CCK8; MedChemExpress), diluted 1:10 in cell culture medium, was added per well and incubated for 1 hour in a humidified incubator at 37 °C. The optical density (OD) value was detected by BIO-RAD iMark™ Microplate Reader at 450 nm.

RNA sequencing in hES-MSCs after *SCDAL* knockdown

Total RNA was isolated from hES-MSCs transfected with *SCDAL* shRNA (n=3) or shRNA control lentiviruses (n=3). Sequencing libraries were generated from the rRNA-depleted RNA using NEBNext® Ultra™ RNA Library Prep Kit for Illumina®. Libraries were sequenced on an Illumina platform by Novogene and 125 bp/150 bp paired-end reads were generated. Clean data (clean reads) were obtained by removing reads containing adapter, reads containing ploy-N and low quality reads from raw data, and then aligned against the *homo sapiens* GRCh38.p12 genome using Hisat2 (v2.0.5)[12]. The mapped reads of each sample were assembled by StringTie (v1.3.3b)[13]. FeatureCounts v1.5.0-p3[14] was used to count the reads numbers mapped to each gene, and then FPKM of each gene was calculated based on the length of the gene and reads count mapped to this gene. Differential expression analysis of two groups was performed using the DESeq2 R package (1.16.1)[15]. The resulting *P* values were adjusted using the Benjamini-Hochberg method for controlling the false discovery rate. Genes with an adjusted *P* value <0.05 found by DESeq2 were assigned as differentially expressed. GO as well as Kyoto Encyclopedia of Genes and Genomes (KEGG) enrichment analysis of differentially expressed genes was implemented by the clusterProfiler R package, in which gene length bias was corrected. GO terms and KEGG pathways with corrected *P* value <0.05 were considered significantly enriched by differential expressed genes.

GDF6 ELISA

GDF6 levels in the cell lysates and conditioned supernatants of culture cells were quantitatively determined by a Human GDF6 ELISA Kit (mlbio, China) following the manufacturer's instructions.

Structural modeling

The protein-protein docking experiments were performed with the structures of human/mouse GDF6 (h/mGDF6) and the extracellular domains of human/mouse VEGFR2 (h/mVEGFR2) using ZDOCK (http://zdock.umassmed.edu/). h/mGDF6 structures were constructed using I-TASSER (https://zhanglab.ccmb.med.umich.edu/I-TASSER/). The structure of hVEGFR2 extracellular domain (20-764) was obtained from RCSB PDB (https://www.rcsb.org/structure/3V2A). The structure of mVEGFR2 extracellular domain (20-762) was homologous modeling constructed using SWISS-MODEL (https://swissmodel.expasy.org/interactive) and hVEGFR2 extracellular domain as a template.

Adhesion frequency assay

The adhesion frequency assay was conducted as described previously[16]. Before assay, HUVECs infected with lentiviruses expressing VEGFR2 or lentiviruses containing empty vectors were characterized by FACS with VEGFR2-PE antibody (Table S2). Briefly, human red blood cells (RBCs) were biotinylated with a biotinylation ester (Jenkem technology) and coated with recombinant human GDF6 protein (abcam,

Cat# ab245811) via biotin-streptavidin coupling, and the RBCs and HUVECs were suspended in DMEM with 1% BSA. Single indicated HUVEC and RBC were respectively aspirated with micropipettes and driven in and out of contact with controlled duration (2 seconds) by a computer-programmed piezoelectric actuator. Adhesion was observed from stretching of the RBC on HUVEC retraction. Four to six pairs of cells were tested, and the contact-retraction cycle was repeated 50 times for each pair of cells.

RNA pull-down and mass spectrometry

Biotinylated *SCDAL* sense and antisense were transcribed in vitro using the T7 RNA polymerase and Biotin RNA Labeling Mix (Roche) and then purified according to the manufacturers' instructions. Biotinylated RNA was incubated with streptavidin Dynabeads, and then incubated with hES-MSC lysates for 2 hours. The samples were separated via electrophoresis and identified using mass spectrometry and retrieved in human proteomic library.

RIP

RIP experiments were performed with the Magna RIP™ RNA-Binding Protein Immunoprecipitation Kit (Merck Millipore) as directed by the manufacturer's instructions. Briefly, ~2×10^7 hES-MSCs were lysed thoroughly on ice in RIP Lysis Buffer supplemented with protease inhibitor cocktail and RNase inhibitor, and the supernatants were collected via centrifugation at 14000 rpm for 10 minutes. 10 μg of

SNF5, BRG1 and BRM antibodies (Table S2) or corresponding IgG were respectively incubated with magnetic beads at room temperature for 30 minutes for each immunoprecipitation, and then the supernatants were incubated with indicated antibody-beads complexes overnight at 4 ^{o}C with rotating. Co-immunoprecipitated RNAs were extracted, reverse-transcribed to cDNA, and subjected to qPCR examination of *SCDAL* enrichment using the primers listed in Table S4.

ChIP-qPCR

ChIP assays were performed using the SimpleChIP® Plus Sonication Chromatin IP Kit (Cell Signaling Technology) and followed the manufacturer's instructions. Briefly, >2×10^7 cells were crosslinked with 1% formaldehyde for 10 minutes, and quenched with glycine for 5 minutes at room temperature. Cell pellets were collected, lysed in ChIP Sonication Cell Lysis Buffer and ChIP Sonication Nuclear Lysis Buffer, and then sonicated with SCIENTZ-IID Ultrasonic Homogenizer (Ningbo Scientz Biotechnology CO,. LTD, China) to generate 500 to 800 bp fragments. Fragmented chromatin was centrifuged at 21000 g for 10 minutes and 200 μl of the supernatant was diluted 1:4 in ChIP buffer to the final volume of 1 ml for each immunoprecipitation reaction. Immunoprecipitation was performed using 10 μg of target antibodies (Table S2) or normal IgG control overnight at 4 ^{o}C with rotating, followed by incubation with ChIP-Grade Protein G Magnetic Beads for 3-4 hours at 4 ^{o}C with rotating. After washing by low salt and high salt buffer, the chromatin fragments were harvested with the elution buffer, and the ChIP eluates were

reverse-crosslinked at 65 ^{o}C for 2 hours with NaCl and Proteinase K. ChIP DNA was purified, and genomic regions of the transcription start sites for the indicated genes were amplified via qPCR with the corresponding primers (Table S4).

Luciferase reporter assay

The indicated region of promoter was cloned into GV238 luciferase reporter plasmid (GENECHEM). Indicated cells cultured in 24-well plates were cotransfected with luciferase reporters, indicated factors and the internal control *Renilla* vector with Lipofectamine® 3000 (Thermo Fisher Scientific, Invitrogen). 48-72 hours later, cell lysates were harvested and luciferase activities were examined using the Dual-Luciferase Reporter Assay System (Promega). Briefly, cultured cells per well were rinsed in PBS, and lysed by gentle shaking in 100 μl 1× Passtive Lysis Buffer for 15 minutes. 20 μl of cell lysates were used to measure firefly luciferase activity by adding 100 μl Luciferase Assay Reagent II and further measure *Renilla* luciferase activity after dispensing 100 μl Stop & Glo® Reagent with GloMax® 20/20 Luminometer (Promega). Firefly luciferase activity was normalized to *Renilla* activity and presented as relative luciferase activity.

Animal experiments

Acute MI model, cell transplantation and lentivirus delivery

Male adult C57BL/6J mice were purchased from Shanghai Slac Laboratory Animal Technology Corporation (China), fed a standard laboratory diet, and maintained with

a 12:12-hour light/dark cycle. Acute MI was surgically induced as described previously[2a], and all surgical procedures were performed by experienced technicians in a blinded manner. Briefly, mice (8-10 weeks old, 20-25 g body weight) were anesthetized by intraperitoneal injection of pentobarbital sodium (60 mg/kg), ventilated via tracheal intubations connected to a rodent ventilator, subjected to MI by permanent ligation of the left anterior descending (LAD) coronary artery using an 8-0 nylon suture. Sham-operated mice underwent all surgical procedure for MI induction except LAD ligation. For cell transplantation or lentivirus delivery, 1×10^5 indicated hES-MSCs/hBM-MSCs or 1×10^7 TU lenti-GDF6 or lenti-control in 20 μl DMEM or an equal volume of DMEM were directly injected into 4 sites around the MI border zone per mouse after coronary artery ligation.

Cardiac function assessments by echocardiography and hemodynamics

All echocardiography and hemodynamics were performed by skilled technicians using identical settings and analyzed blinded to the experimental status of the animals. Echocardiography was performed on conscious, gently restrained mice with a Vevo 2100 system before MI (baseline) as well as 3, 7, 14 and 28 days after MI. Two-dimensional M-mode images of the left ventricle were captured at the level of the papillary muscles. Left ventricular internal diameters were measured at end-diastole (LVIDd) and end-systole (LVIDs), and then EF and FS were calculated as described previously[17]. Cardiac hemodynamics was measured after the final echocardiographic examination. Briefly, mice were anesthetized with pentobarbital

sodium (60 mg/kg). The right common carotid artery was dissected under a dissecting microscope (SZX7, Olympus) and cannulated with a 1.4-Fr micro-tipped Millar (Millar Instruments). The transducer was connected to a computerized data acquisition system (PowerLab, AD Instruments) to record heart rate, blood pressure, ventricular pressures and ±dp/dt. Baseline zero reference was obtained by placing the sensor in normal saline before insertion.

Triphenyltetrazolium chloride (TTC) staining

Four weeks after MI, the entire hearts were snap-frozen, sectioned into six 1-mm-thick transverse slices from the apex to the base, incubated in a 1% solution of phosphate-buffered TTC (Sigma-Aldrich) at 37°C in the dark for 10 minutes. The infarct area (white) and viable myocardium (brick red) were delineated and calculated using Image-Pro Plus software 6.0. The ratio of the infarct area to the LV area was calculated and presented as a percentage.

Fibrotic area determination and immunofluorescence staining

Heart tissues were respectively harvested 3 days after MI to measure the survival of transplanted cells, cardiac apoptosis, inflammation and endothelial proliferation, or 28 days after MI to evaluate fibrotic area and vessel density. They were either dehydrated in 30% sucrose solution, embedded in Tissue-Tek OCT compound (SAKURA) and snap frozen in liquid nitrogen, or fixed in 10% formalin and paraffin-embedded. The fibrotic area in the left ventricle was detected by Sirius Red staining and Masson

staining. Paraffin-embedded heart sections (3.0 μm thick) were deparaffinized in xylene, rehydrated through graded alcohols to distilled water, and stained with Sirius Red (Solarbio LIFE SCIENCES, China) for 30 minutes followed by washing with 95% ethyl alcohol. Frozen heart sections (6.0 μm thick) were stained with Masson's trichrome kit (MXB Biotechnologies, China). The percentage of fibrotic area was calculated as the mean value of the endocardial and epicardial length of the whole fibrotic area in proportion to the mean length of the endocardial and epicardial left ventricle using Image-Pro Plus software 6.0.

For immunofluorescence staining, frozen heart sections were fixed with 10% formalin for 10 minutes, permeabilized with 0.2% Triton X-100 for 15 minutes, blocked with 10% bovine serum albumin for 1 hour, incubated overnight at 4 ^{o}C with primary antibodies against GFP, CD31, SMA, Troponin, Ki67, CD68 and CD3 (Table S2), and then incubated with the corresponding secondary antibodies for 1 hour at room temperature. Staining without the primary antibodies was used as control for nonspecific fluorescence. Nuclei were counterstained with Hoechst 33258, and sections were imaged under a Leica fluorescent microscope. Capillary and arteriole density were respectively quantified as the number of CD31^{+} vessels per HRF or the number of SMA^{+} vessels per HRF in the border, infarct and remote areas of hearts. Endothelial proliferation was quantified as the number of CD31^{+}/Ki67^{+} cells per HRF, and inflammation was quantified as the number of CD68^{+} and CD3^{+} cells per HRF. For assessments of apoptosis, frozen and fixed sections were detected by TUNEL staining (In Situ Cell Death Detection Kit, TMR red; Roche Applied Science).

Cardiac apoptosis was quantified as the percentage of $TUNEL^+$ nuclei per HRF in the border area of hearts, whereas apoptotic transplanted hES-MSCs/hBM-MSCs were quantified as the percentage of $TUNEL^+/GFP^+$ cells per HRF.

Data availability

The RNA sequencing data used in the study are available in a public repository from NCBI. The accession numbers are SRP192798 for the lncRNA sequencing data, and SRP192797 for the mRNA sequencing data.

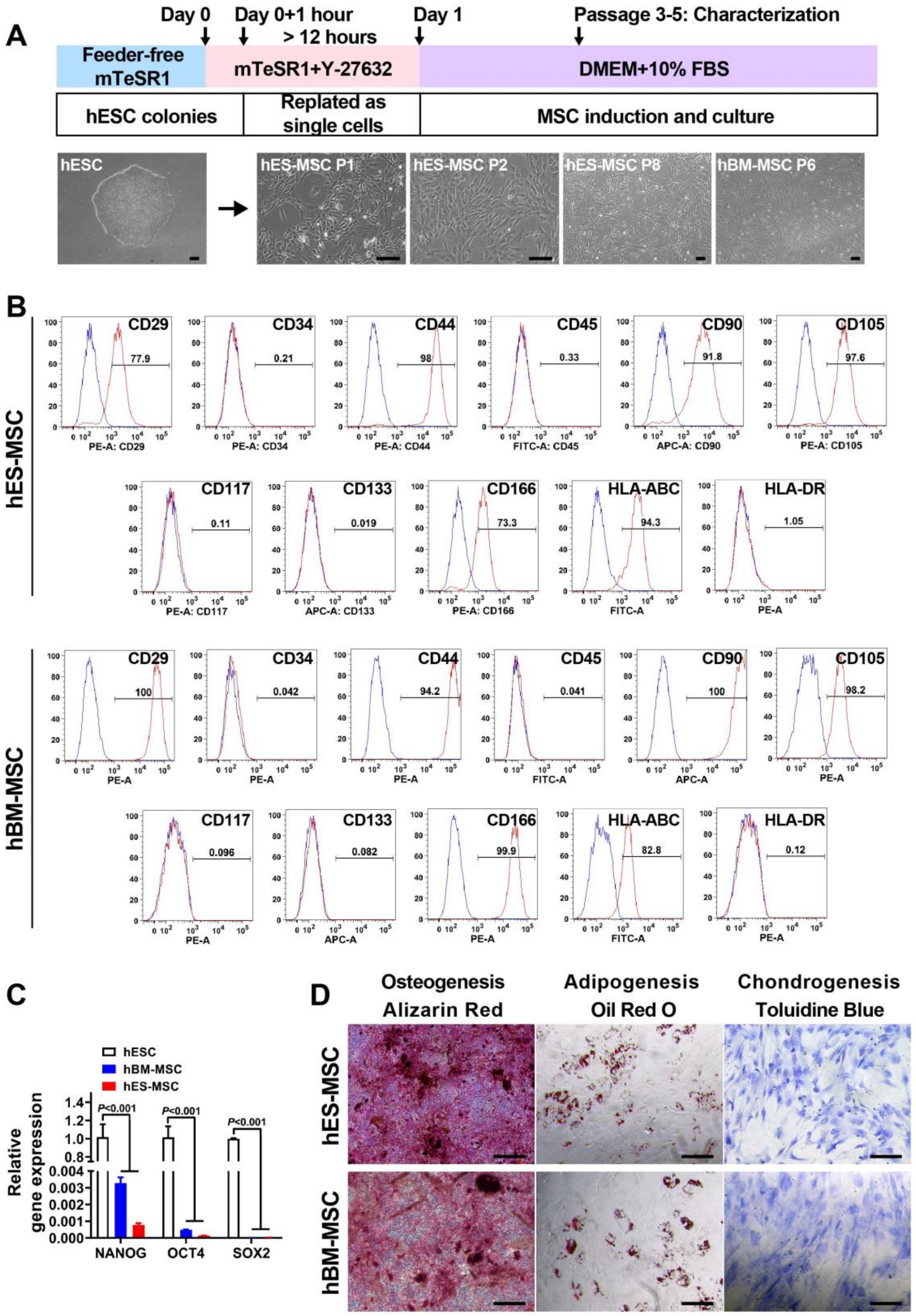

Figure S1. Derivation and characterization of hES-MSCs. (A) Schematic protocol

for the derivation of MSCs from hESCs. Phase-contract images of cultured hESC colony before differentiation and induced hES-MSCs at different passages. Cultured hBM-MSCs are shown as comparison. Scale bar, 200μm. (B) Flow cytometry analysis of surface markers in hES-MSCs and hBM-MSCs. Red histograms display percent cells expressing the indicated surface antigens. Blue histograms represent the isotype controls. (C) Relative expression of pluripotent genes in hESCs, hBM-MSCs and hES-MSCs by qRT-PCR. All bars represent mean ± SEM (n=3, One-way ANOVA, LSD, S-N-K, and Waller-Duncan analysis). (D) Multilineage differentiation of hES-MSCs and hBM-MSCs. The differentiated cells are stained for osteogenic mineralization with Alizarin Red, for lipid droplets with Oil Red O, or for chondrocytes with Toluidine Blue, respectively. Scale bar, 200μm.

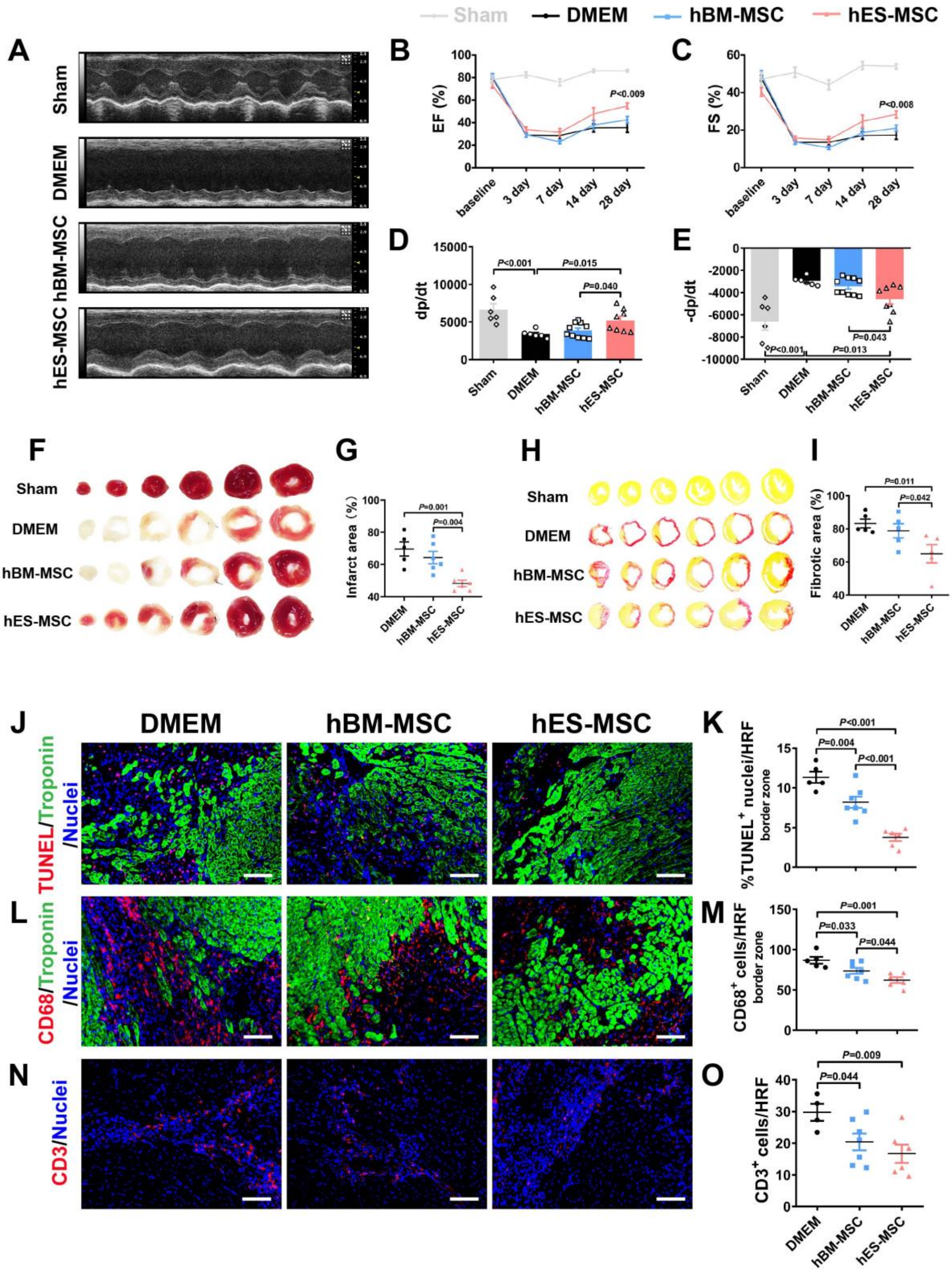

Figure S2. Comparison of hES-MSC and hBM-MSC transplantation after MI.

(A) Representative M-mode tracings from Sham, DMEM, hBM-MSC and

hES-MSC-receiving mice 28 days post MI. (B-C) Sequential echocardiographical assessments of LV EF and FS in Sham (n=9), DMEM (n=15), hBM-MSC (n=15) and hES-MSC (n=13)-receiving mice at baseline as well as 3, 7, 14 and 28 days after transplantation. (D-E) Hemodynamic assessments of ±dp/dt four weeks post MI in Sham (n=6), DMEM (n=6), hBM-MSC (n=10) and hES-MSC (n=8)-receiving mice. (F-I) Infarct area and fibrotic area quantification respectively by TTC staining and Sirius Red staining on serial heart sections four weeks post MI in Sham, DMEM (n=5), hBM-MSC (n≥5) and hES-MSC (n≥5)-receiving mice. (J-O) Representative images and quantification of $TUNEL^+$ nuclei (J-K), $CD68^+$ (L-M) and $CD3^+$ cells (N-O) three days after MI in mouse hearts transplanted with DMEM (n≥4), hBM-MSCs (n=7) and hES-MSCs (n=6). Scale bar, 100µm. HRF, high resolution field. All bars in B-E, G, I, K, M and O represent mean ± SEM (One-way ANOVA, LSD, S-N-K, and Waller-Duncan analysis).

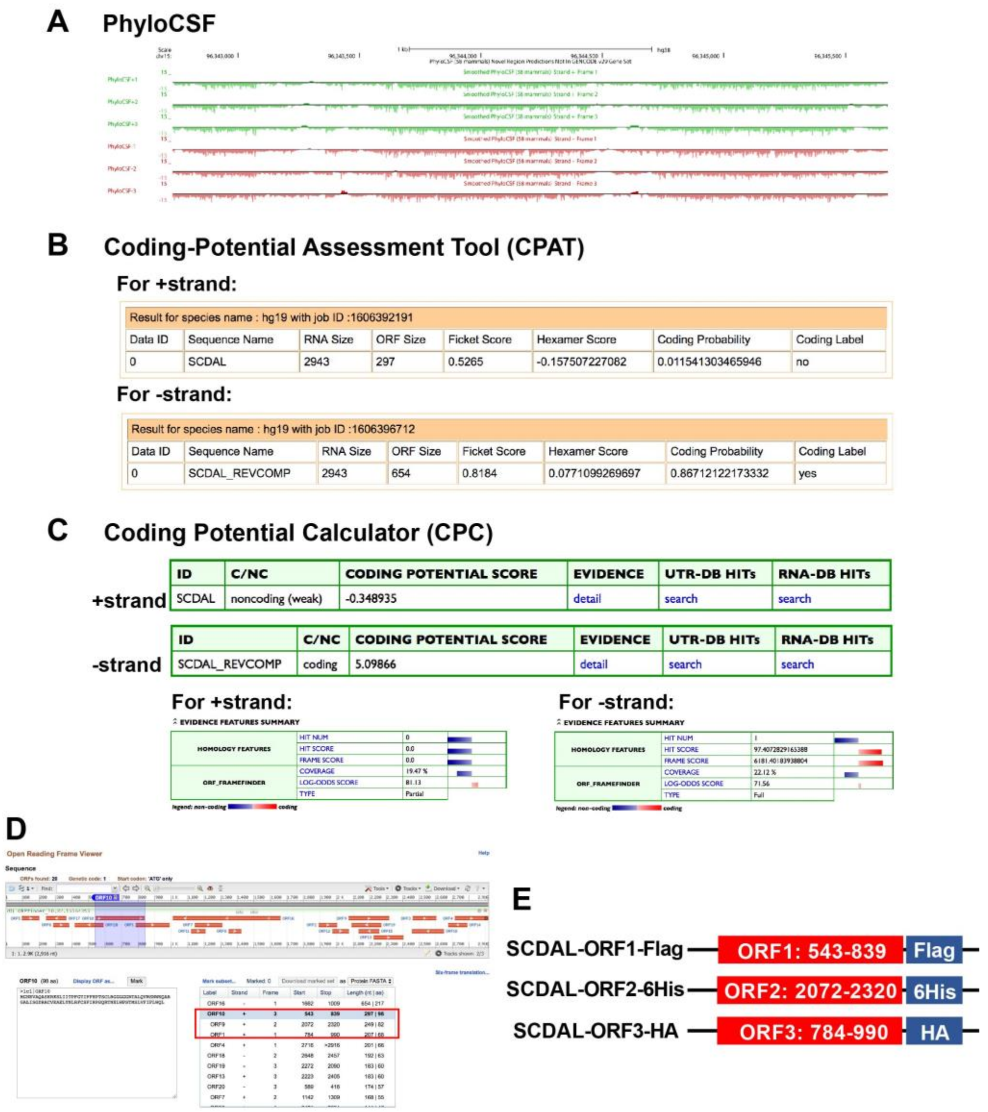

Figure S3. Protein-coding potential predication of *SCDAL*. (A-C) *SCDAL* protein-coding potential is respectively predicted with PhyloCSF, Coding-Potential Assessment Tool (CPAT) and Coding Potential Calculator (CPC). PhyloCSF gives negative scores for plus and minus strands of *SCDAL*, indicating that *SCDAL* does not have protein-coding potential. CPAT and CPC give “noncoding” for plus strand of *SCDAL*, but “coding” for minus strand of *SCDAL*. (D) Predication of *SCDAL* ORFs by NCBI ORFfinder. Red rectangle indicates three most potentially translated ORFs.

(E) Diagram of the *SCDAL*-ORF1-Flag, *SCDAL*-ORF2-6His and *SCDAL*-ORF3-HA fusion constructs used for transfection.

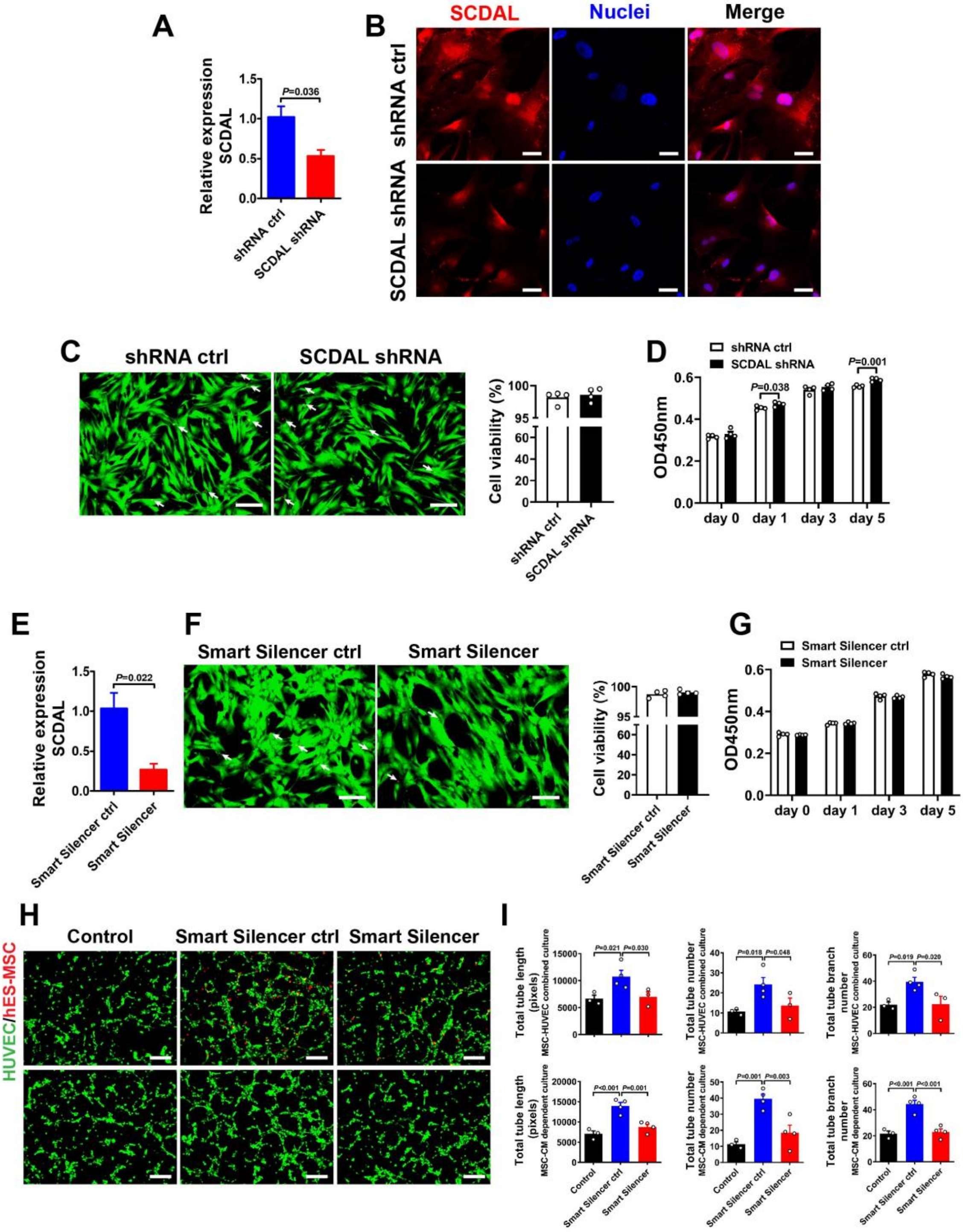

Figure S4. Cell viability and angiogenic potential of *SCDAL* depleted hES-MSCs. (A) *SCDAL* expression in hES-MSCs after lentiviral shRNA transfection (*SCDAL* shRNA, n=3). (B) FISH confirmation of decreased *SCDAL* expression in *SCDAL* shRNA hES-MSCs compared to shRNA ctrl hES-MSCs. Scale bar, 50μm. (C-D) Cell viability and proliferation of hES-MSCs after *SCDAL* knockdown (n=4). Scale bar, 200μm. White arrows indicate dead cells. (E) *SCDAL* expression in hES-MSCs after transfection of Smart Silencer (n=3). (F-G) Cell viability and proliferation of hES-MSCs after Smart Silencer transfection (n=4). Scale bar, 200μm. White arrows indicate dead cells. (H-I) Representative images and quantification for HUVEC tube formation (green) cocultured with Smart Silencer-transfected hES-MSCs (red, top panels) or their conditioned media (bottom panels) as compared to control groups (n≥3). Scale bar, 200μm. All bars in A, C-G and I represent mean ± SEM (A and C-G, unpaired Student's *t* test; I, One-way ANOVA, LSD, S-N-K, and Waller-Duncan analysis).

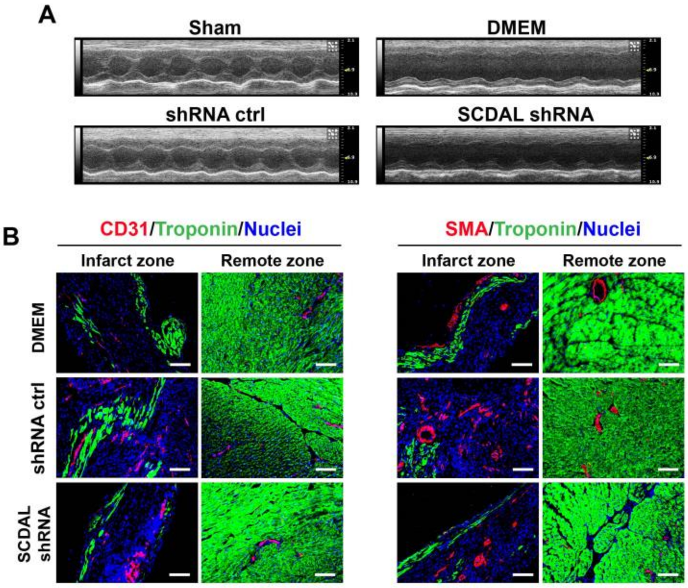

Figure S5. Cardiac function and angiogenesis after *SCDAL* depleted hES-MSC transplantation. (A) Representative M-mode images of the LV in Sham, DMEM, shRNA ctrl hES-MSC and *SCDAL* shRNA hES-MSC-receiving mice 28 days after MI. (B) Representative infarct and remote zone images of $CD31^{+}$ capillaries (left panels) and SMA^{+} arterioles (right panels) in different transplanted hearts four weeks after MI. Scale bar, 100μm.

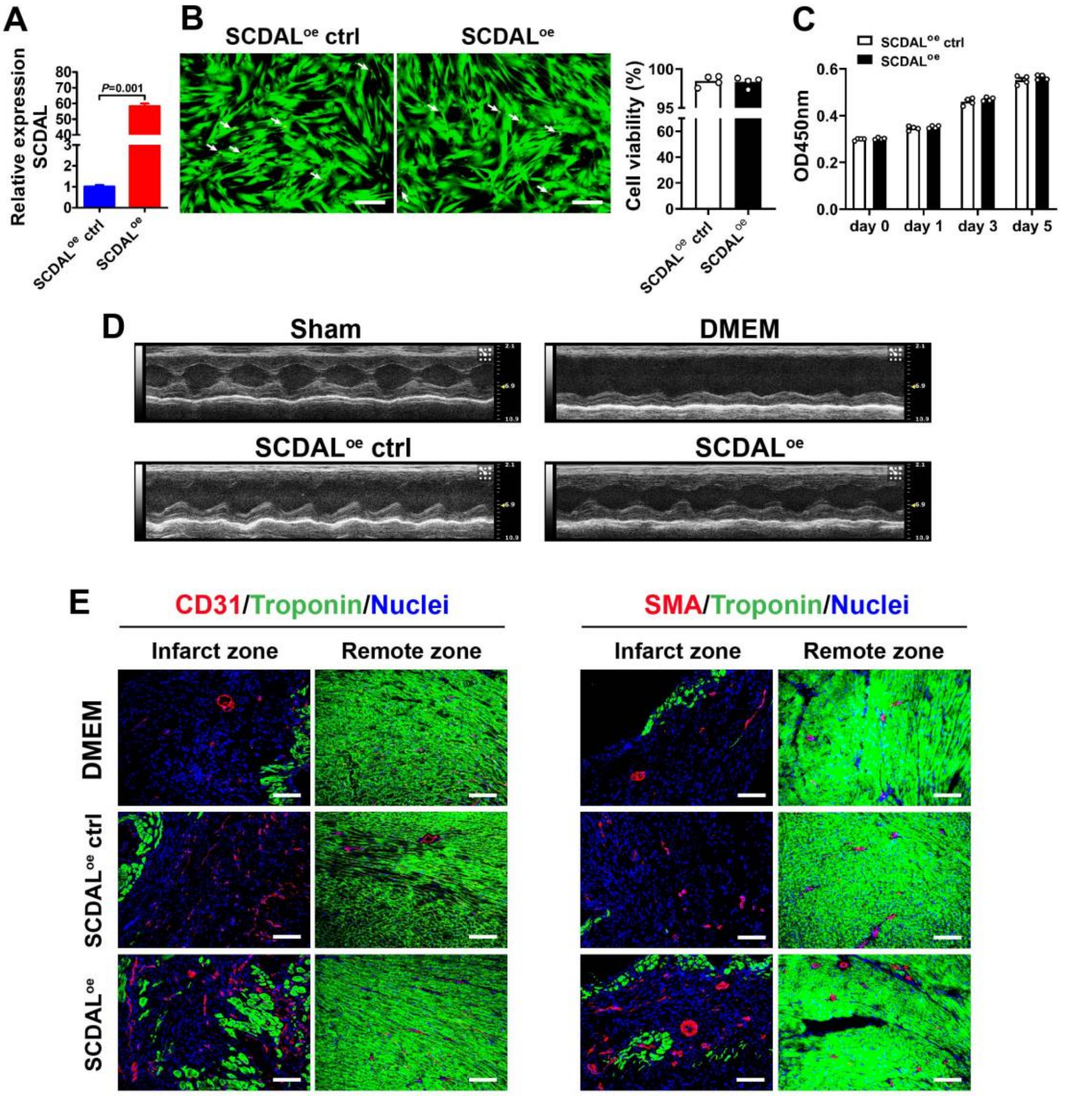

Figure S6. Cardiac function and angiogenesis after *SCDAL* overexpressing hES-MSC transplantation. (A) *SCDAL* expression in hES-MSCs after lentiviral overexpression (*SCDAL*^{oe}, n=3). (B-C) Cell viability and proliferation of hES-MSCs after *SCDAL* overexpression (n=4). Scale bar, 200μm. White arrows indicate dead cells. (D) Representative M-mode images of the LV four weeks post MI in Sham, DMEM, *SCDAL*^{oe} ctrl hES-MSC and *SCDAL*^{oe} hES-MSC-receiving mice. (E) Representative infarct and remote zone images of CD31^{+} (left panels) and SMA^{+}

(right panels) vessels in different transplanted hearts four weeks post infarction. Scale bar, 100μm. All bars in A-C represent mean ± SEM (unpaired Student's *t* test).

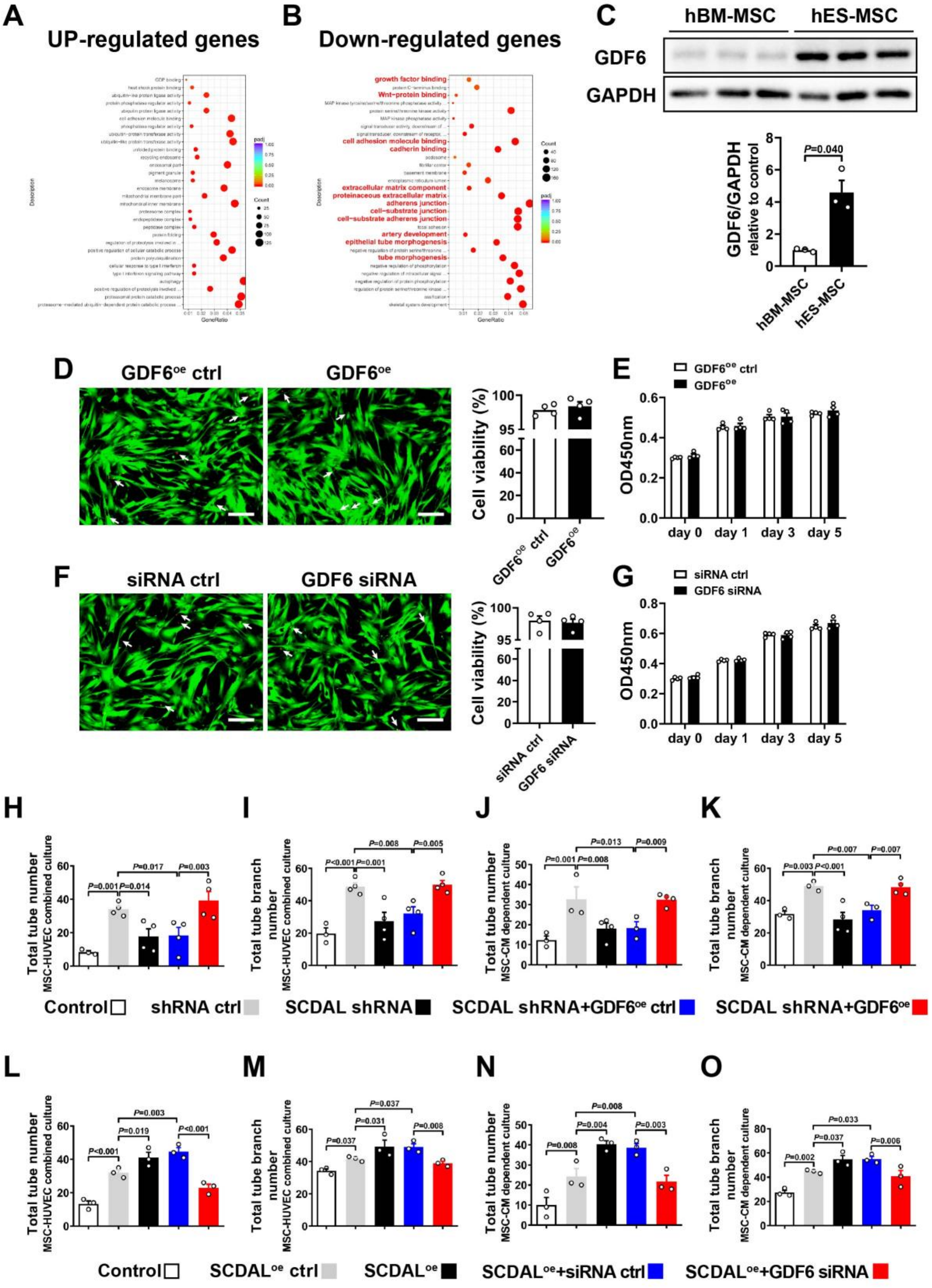

Figure S7. GO analysis of the differentially expressed genes in hES-MSCs

following *SCDAL* depletion. (A-B) GO terms linked to biological processes for the up-regulated or down-regulated genes in *SCDAL* depleted hES-MSCs. (C) Quantification of GDF6 protein levels between hBM-MSCs and hES-MSCs following western blot detection (n=3). (D-G) Cell viability and proliferation of hES-MSCs after GDF6 overexpression or depletion (n=4). Scale bar, 200μm. White arrows indicate dead cells. (H-O) Quantification for total tube number and tube branch number of HUVECs cocultured with indicated hES-MSCs or their supernatants from *SCDAL* knockdown GDF6 rescue and *SCDAL* overexpression GDF6 depletion (n≥3). All bars in C-O represent mean ± SEM (C-G, unpaired Student's *t* test; H-O, One-way ANOVA, LSD, S-N-K, and Waller-Duncan analysis).

A

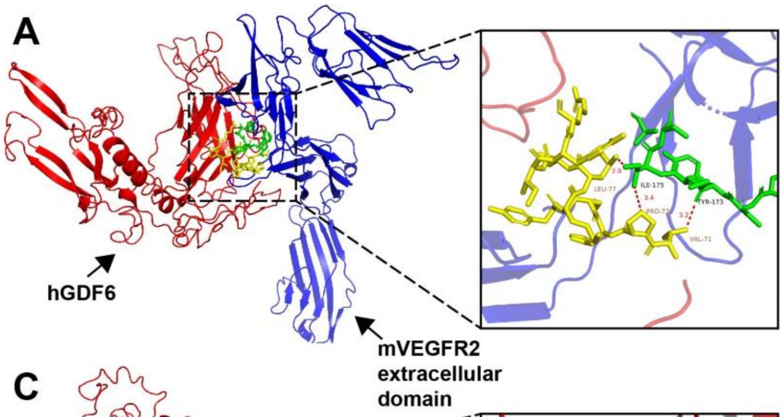

B

hGDF6	mVEGFR2 extracellular domain	Distance
LEU-77	ILE-175	2.0
PRO-72	ILE-175	3.4
VAL-71	TYR-173	3.2

C

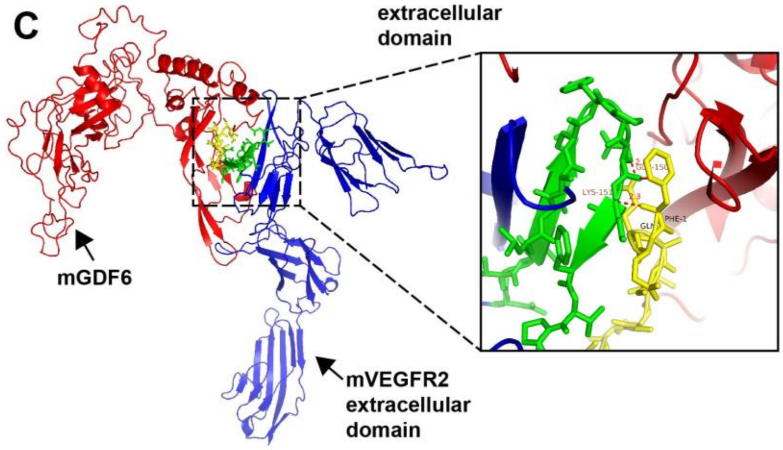

D

mGDF6	mVEGFR2 extracellular domain	Distance
GLN-3	GLU-150	2.8
PHE-1	LYS-151	2.3
PHE-1	GLU-150	2.5

E Alignment of VEGFR2 extracellular domain

```
hGDF6: mVEGFR2  -------------------ASVGLPGDFLHPPKLSTQKDILTILANTTLQITCRGQRDLD
mGDF6: mVEGFR2  -------------------ASVGLPGDFLHPPKLSTQKDILTILANTTLQITCRGQRDLD
hGDF6: hVEGFR2  MQSKVLLAVALWLCVETRAASVGLPSVSLDLPRLSIQKDILTIKANTTLQITCRGQRDLD
                                   ******.  *  *.** ******* ****************

hGDF6: mVEGFR2  WLWPNAQRDSEERVLVTECGGGDSIFCKTLTIPRVVGNDTGAYKCSYRDVDIASTVYVYV
mGDF6: mVEGFR2  WLWPNAQRDSEERVLVTECGGGDSIFCKTLTIPRVVGNDTGAYKCSYRDVDIASTVYVYV
hGDF6: hVEGFR2  WLWPNNQSGSEQRVEVTECSDG--LFCKTLTIPKVIGNDTGAYKCFYRETDLASVIYVYV
                ***** * .**:** ****..*  :*********.*:*********  **:.*:**.:****

hGDF6: mVEGFR2  RDYRSPFIASVSDQHGIVYItenknkTVVIPCRGSISNLNVSLCARypEKRFVPDGNRIS
mGDF6: mVEGFR2  RDYRSPFIASVSDQHGIVYITeNKNKTVVIPCRGSISNLnVSlcarypekrfvpDGNRIS
hGDF6: hVEGFR2  QDYRSPFIASVSDqhgvvyITENknKTVvipclGSISNLnVslcarypekrfvpdGnriS
                .****************:************** ****************************
                                                    Ig-like domain 2

hGDF6: mVEGFR2  WDSEIGFTLPsymisyaGMVFCEAKINDETYQSIMYIVvvvgYRIydviLSPPHEIELSA
mGDF6: mVEGFR2  WDSEIGFTLPSymisyagmvfCeAKINDETYQSIMyivvvvGYRIYDVILSPPHEIELSA
hGDF6: hVEGFR2  WDSKkgfTipSymisyagmVfceakiNdesyqsimyiVvVVGYRIYDVVLSPSHGIELSV
                ***: ***:**************************:*****************:***.* ****.
                Ig-like domain 2                                Ig-like domain 3

hGDF6: mVEGFR2  GEKLVLnCtartelnvGLDFTWHSPPSKSHHKkIVnrdvkpfpgtvakmflSTLTIESVT
mGDF6: mVEGFR2  GEKLVLNCTARTelnVGLDFTWHSPPSKSHHKKIVNRDVkpfpgtvakMFLSTLTIESVT
hGDF6: hVEGFR2  GEKLVLNCTARTELNVgidfnweYPSSKHQHKKlVnRDLKTQSGSEMKKFLSTLTIDGVT
                *****************:**.*  *.** :***:****:*. .*:  * *******:.**
                                  Ig-like domain 3
```

Figure S8. Interface between GDF6 and VEGFR2. (A, C) h/mGDF6 (red) binding interface on mVEGFR2 extracellular domain (blue). The boxed regions show the binding sites, with h/mGDF6 colored in yellow and mVEGFR2 extracellular domain colored in green. (B, D) The tables show the key interacting residues between h/mGDF6 and mVEGFR2 extracellular domain. (E) Sequence alignment of VEGFR2 extracellular domains from human and mouse, with Ig-like domain 2 and 3 boxed in yellow and green. The binding sites of hGDF6 on hVEGFR2, hGDF6 on mVEGFR2 and mGDF6 on mVEGFR2 are highlighted in red and orange.

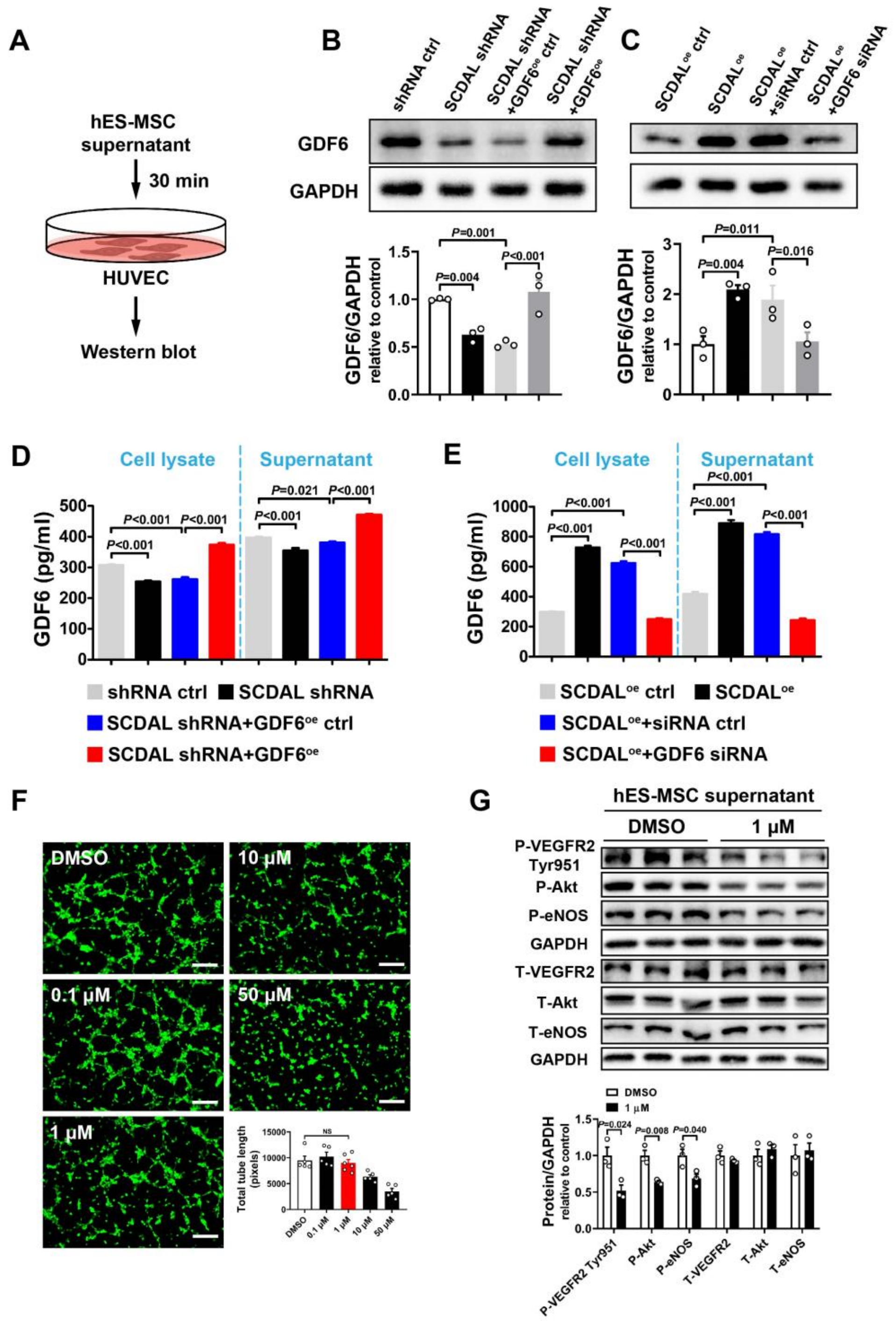

Figure S9. Measurement of *SCDAL*-mediated GDF6 secretion and selection of optimal dose of ZM323881. (A) Analysis of signaling activation in HUVECs after incubated with hES-MSC-derived conditioned supernatants. (B-C) Western blot (n=3) and (D-E) ELISA (n=3) quantification of GDF6 expression and secretion in different

hES-MSC groups of (B, D) *SCDAL* knockdown GDF6 rescue and (C, E) *SCDAL* overexpression GDF6 depletion. (F) Representative images and quantification of HUVEC tube formation under basal condition after treated with increasing doses of ZM323881 (n≥5). Scale bar, 200μm. NS, no significance. (G) Quantification of hES-MSC supernatant-induced VEGFR2/Akt/eNOS activation in HUVECs in the presence or absence of 1 μM ZM323881 (n=3). DMSO serves as a solvent control. All bars in B-G represent mean ± SEM (B-F, One-way ANOVA, LSD, S-N-K, and Waller-Duncan analysis; G, unpaired Student's *t* test).

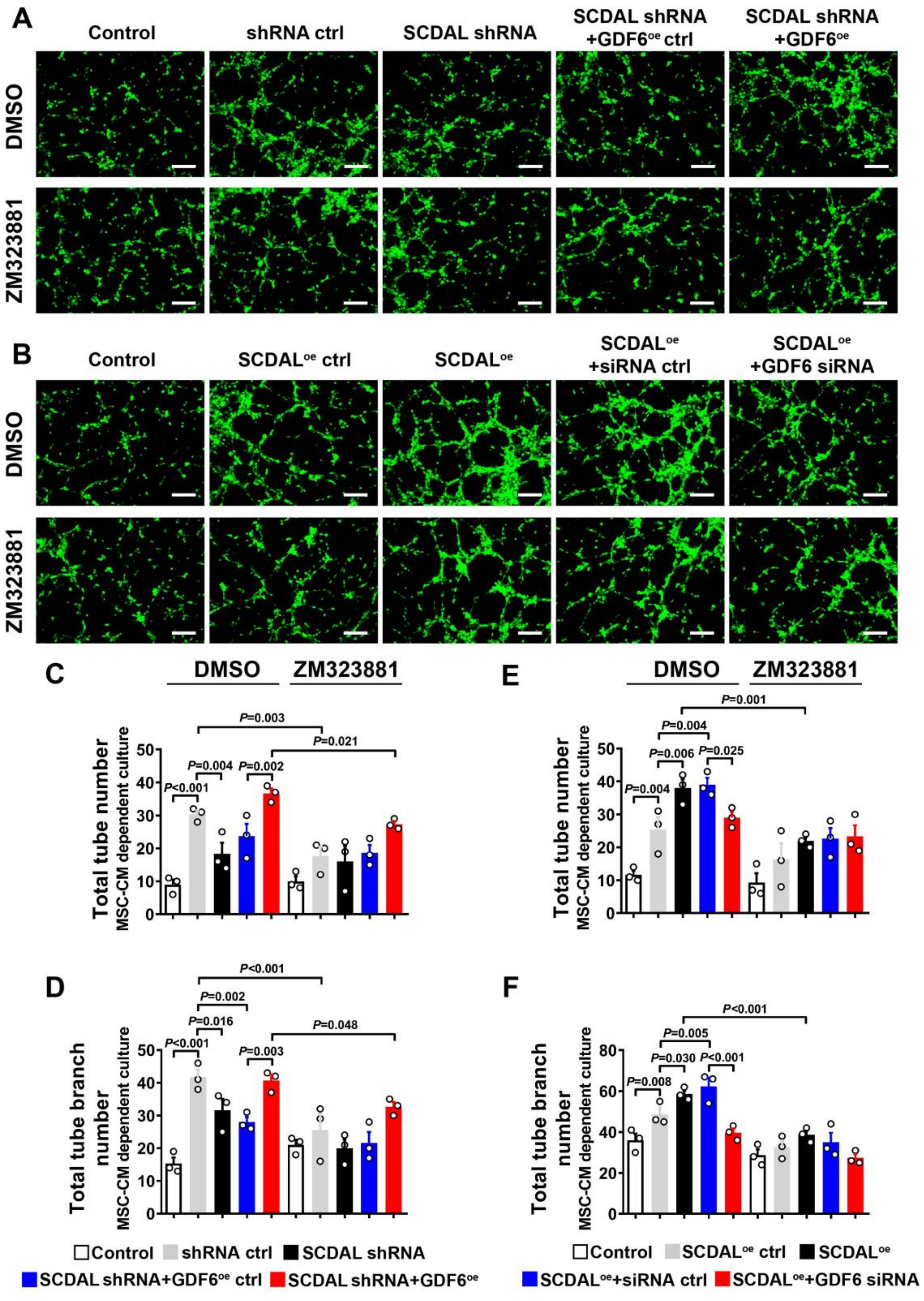

Figure S10. ***SCDAL*-induced GDF6 secretion promotes endothelial angiogenesis**

via VEGFR2. (A-B) Representative HUVEC tube formation images and (C-F) corresponding quantification for total tube number and tube branch number under different hES-MSC supernatants of (A, C, D) *SCDAL* knockdown GDF6 rescue and (B, E, F) *SCDAL* overexpression GDF6 depletion with or without VEGFR2 blocking (ZM323881) (n=3). DMSO serves as a solvent control. Scale bar, 200μm. All bars in C-F represent mean ± SEM (One-way ANOVA, LSD, S-N-K, and Waller-Duncan analysis).

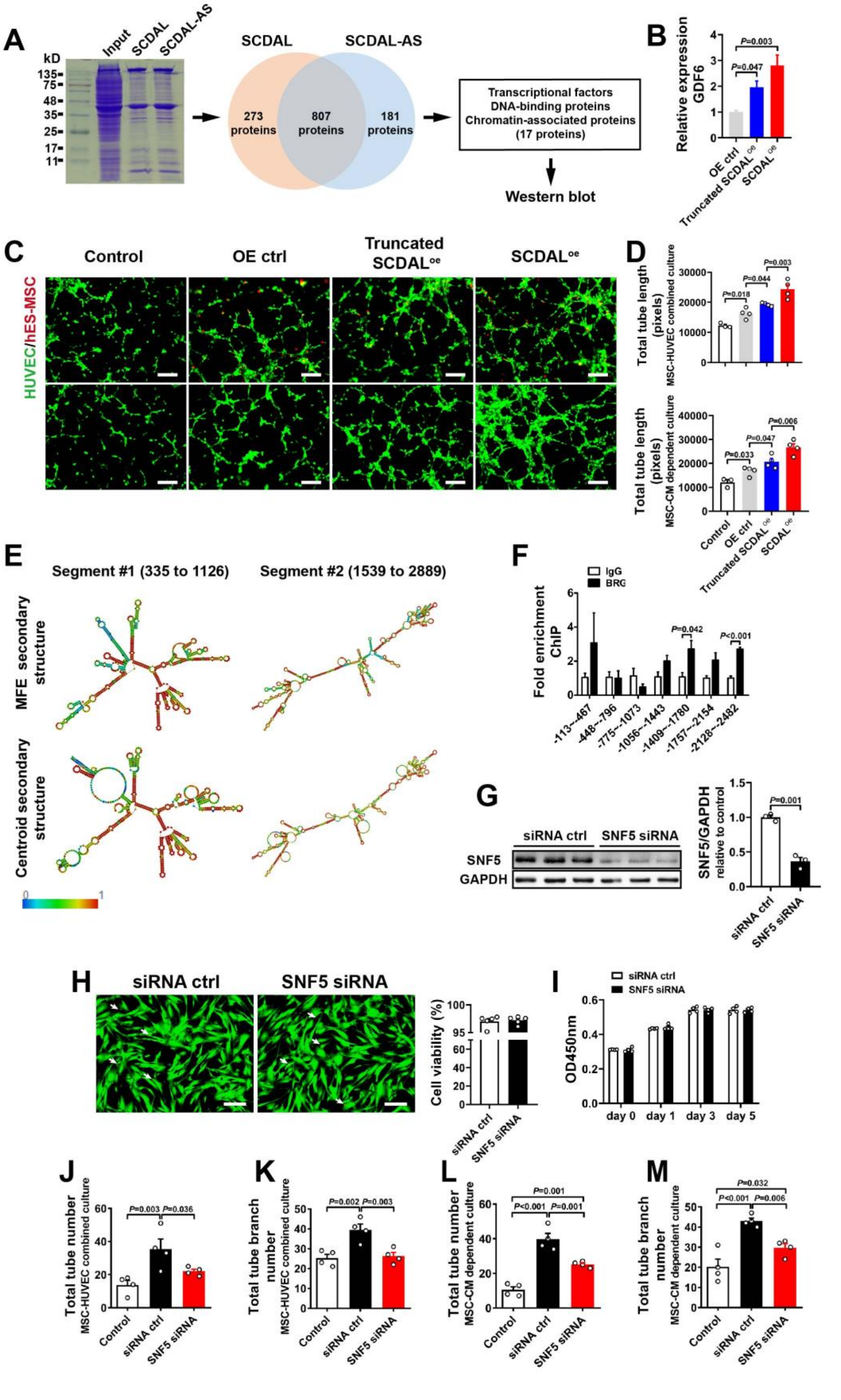

Figure S11. ***SCDAL*** **is associated with SNF5 to promote angiogenesis.** (A)

Full-length *SCDAL* and its antisense (*SCDAL-AS*) are labelled with biotin and incubated with hES-MSC lysates, followed by coomassie blue staining and mass spectrometry. Selection strategy of *SCDAL*-associated proteins from mass spectrometry data. (B) GDF6 expression in hES-MSCs after overexpression of truncated and full-length *SCDAL* (n=3). (C-D) Representative images and quantification of HUVEC (green) tube formation cocultured with truncated $SCDAL^{oe}$ and $SCDAL^{oe}$ hES-MSCs (red, top panels) or their conditioned supernatants (bottom panels) as compared to control groups (n≥3). (E) RNA folding analysis of *SCDAL* segments by RNAfold WebServer (http://rna.tbi.univie.ac.at/cgi-bin/RNAWebSuite/RNAfold.cgi). Predictions are based on minimum free energy (MFE) and partition function. Color scales denote confidence of predictions for each base with shades of red indicating strong confidence. (F) ChIP-qPCR examination of BRG1 binding on the GDF6 promoter in hES-MSCs (n=3). (G) Quantification of SNF5 knockdown efficiency in hES-MSCs after siRNA transfection compared to control (n=3). (H-I) Cell viability and proliferation of hES-MSCs after SNF5 knockdown (n≥4). Scale bar, 200μm. White arrows indicate dead cells. (J-M) Quantification for total tube number and tube branch number of HUVECs after cocultured with SNF5 depleted hES-MSCs or their conditioned supernatants as compared to control groups (n=4). All bars in B, D, and F-M represent mean ± SEM (B, D, J-M, One-way ANOVA, LSD, S-N-K, and Waller-Duncan analysis; F-I, unpaired Student's *t* test).

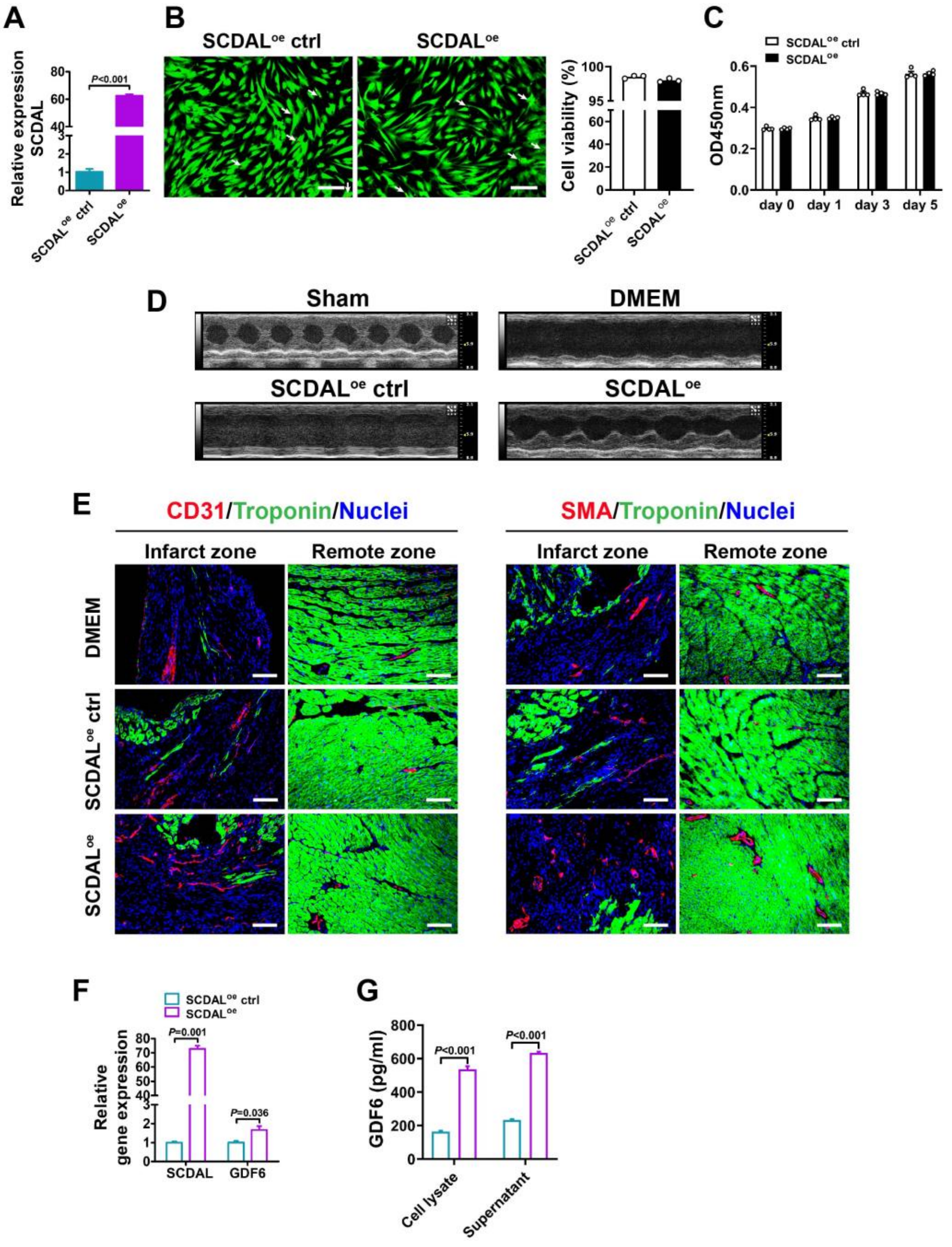

Figure S12. Cardiac function and angiogenesis after *SCDAL* overexpressing hBM-MSC transplantation. (A) *SCDAL* expression in hBM-MSCs after lentiviral overexpression (*SCDAL*^{oe}, n=3). (B-C) Cell viability and proliferation of hBM-MSCs

after *SCDAL* overexpression (n≥3). Scale bar, 200μm. White arrows indicate dead cells. (D) Representative M-mode images of the LV in Sham, DMEM, *SCDAL*^{oe} ctrl hBM-MSC and *SCDAL*^{oe} hBM-MSC-receiving mice 28 days after MI. (E) Representative infarct and remote zone images of CD31^{+} (left panels) and SMA^{+} vessels (right panels) in different transplanted hearts four weeks after MI. Scale bar, 100μm. (F-G) qRT-PCR and ELISA analysis of GDF6 expression and secretion in hBM-MSCs after *SCDAL* overexpression (n=3). All bars in A-C and F-G represent mean ± SEM (unpaired Student's *t* test).

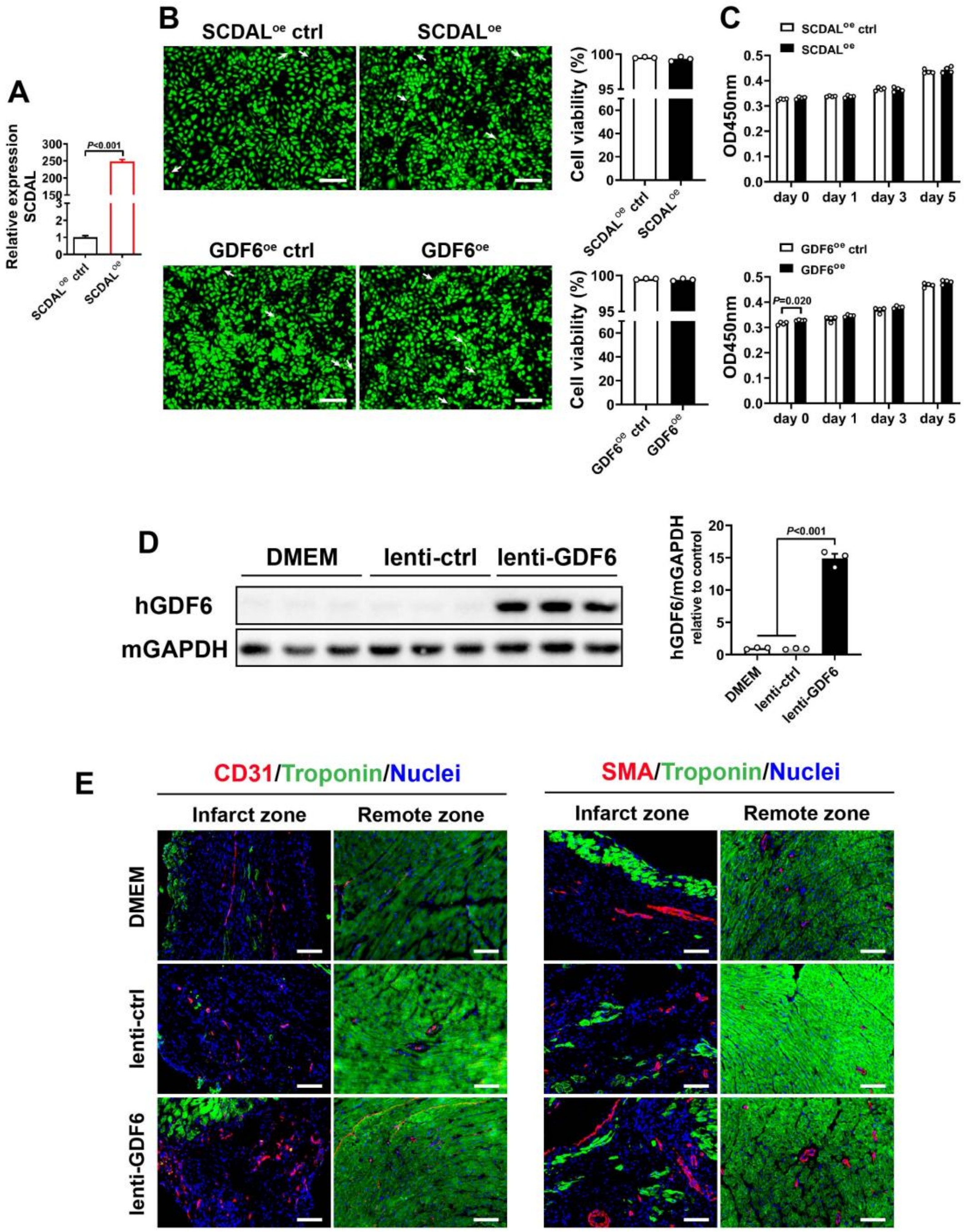

Figure S13. GDF6 overexpression promotes angiogenesis after MI. (A) qRT-PCR confirmation of *SCDAL* overexpression in HUVECs (n=3). (B-C) Cell viability and proliferation of HUVECs after *SCDAL* or GDF6 overexpression (n≥3). Scale bar, 200μm. White arrows indicate dead cells. (D) Quantification of hGDF6

overexpression efficiency in DMEM, lenti-ctrl and lenti-GDF6-receiving mouse hearts (n=3). (E) Representative infarct and remote zone images of $CD31^+$ (left panels) and SMA^+ (right panels) vessels in different transplanted hearts four weeks post infarction. Scale bar, 100μm. All bars in A-D represent mean ± SEM (A-C, unpaired Student's *t* test; D, One-way ANOVA, LSD, S-N-K, and Waller-Duncan analysis).

Table S1. Donor characteristics and passages of hBM-MSCs

	**Age (year)**	**Sex**	**Passage**
Donor #1	62	Female	P3-P6
Donor #2	46	Female	P3-P6
Donor #3	43	Female	P3-P6
Donor #4	45	Male	P5-P8
Donor #5	59	Female	P5-P8
Donor #6	55	Male	P5-P8
Donor #7	51	Female	P5-P8
Donor #8	56	Female	P5-P8
Donor #9	60	Male	P5-P8
Donor #10	63	Male	P5-P8

Table S2. List of the antibodies used in fluorescence activated cell sorting (FACS), immunofluorescence (IF), western blot (WB), RIP and ChIP

Antibodies	Source	Identifier	Application
PE Mouse Anti-Human CD29 (Integrin beta 1)	eBioscience	Cat# 12-0299	FACS
PE Mouse Anti-Human CD34	Miltenyi Biotec	Cat# 130-081-002	FACS
PE Rat Anti-Human/Mouse CD44	eBioscience	Cat# 12-0441	FACS
FITC Mouse Anti-Human CD45	eBioscience	Cat# 11-0459	FACS
APC Mouse Anti-Human CD90 (Thy-1)	eBioscience	Cat# 17-0909	FACS
PE Mouse Anti-Human CD105 (Endoglin)	eBioscience	Cat# 12-1057	FACS
PE Mouse Anti-Human CD117 (c-Kit)	eBioscience	Cat# 12-1178	FACS
APC Mouse Anti-Human CD133 (AC133)	Miltenyi Biotec	Cat# 130-090-826	FACS
PE Mouse Anti-Human CD166	BD Biosciences	Cat# 560903	FACS
FITC Mouse anti-Human HLA-ABC	BD Biosciences	Cat# 560965	FACS
PE-CF594 Mouse Anti-Human HLA-DR	BD Biosciences	Cat# 562331	FACS
PE Mouse anti-human CD309 (VEGFR2)	BioLegend	Cat# 359904	FACS
Rabbit polyclonal to anti-Cardiac Troponin I	Abcam	Cat# 47003	IF
Goat polyclonal to anti-Cardiac Troponin C	Abcam	Cat# ab30807	IF
Rat monoclonal anti-CD31	BD Biosciences	Cat# 550274	IF
Rabbit polyclonal to anti-alpha Smooth Muscle Actin	Abcam	Cat# 5694	IF
Rabbit monoclonal to anti-Ki67	Abcam	Cat# ab16667	IF
Rat monoclonal to anti-CD68	BIO-RAD	Cat# MCA1957T	IF
Rat monoclonal to anti-CD3	Abcam	Cat# ab33429	IF
Rabbit polyclonal to anti-GFP	Abcam	Cat# ab290	IF
Mouse monoclonal to anti-Flag tag	Abcam	Cat# ab49763	WB
Mouse monoclonal to anti-6His tag	proteintech	Cat# 66005-1-lg	WB
Rabbit polyclonal to anti-HA tag	Abcam	Cat# ab9110	WB
Rabbit polyclonal to anti-GDF6	Abcam	Cat# ab226853	WB
Mouse monoclonal to anti-Flk-1 (VEGFR2) (A-3)	Santa Cruz Biotechnology	Cat# sc-6251	WB
Rabbit monoclonal to anti-Phospho-VEGF Receptor 2 (Tyr951) (15D2)	Cell Signaling Technology	Cat# 4991	WB
Rabbit monoclonal to anti-Akt (C67E7)	Cell Signaling Technology	Cat# 4691S	WB
Rabbit monoclonal to anti-Phospho-Akt (Ser473) (D9E)	Cell Signaling Technology	Cat# 4060S	WB
Rabbit polyclonal to anti-eNOS	Cell Signaling Technology	Cat# 9572S	WB
Mouse monoclonal to anti-NOS3 (eNOS) (A-9)	Santa Cruz Biotechnology	Cat# sc-376751	WB
Mouse monoclonal to anti-Phospho-NOS3 (eNOS) (15E2)	Santa Cruz Biotechnology	Cat# sc-81510	WB
Rabbit polyclonal to anti-SNF5	Abcam	Cat# ab12167	WB, RIP, ChIP, IF
Rabbit monoclonal to anti-BRG1	Abcam	Cat# ab110641	WB, RIP, ChIP, IF

Rabbit monoclonal to anti-BRG1	Cell Signaling Technology	Cat# 49360	WB, RIP, ChIP, IF
Rabbit monoclonal to anti-BRM	Cell Signaling Technology	Cat# 11966	RIP
HRP-conjugated mouse monoclonal to anti-GAPDH	KANGCHEN	Cat# KC-5G5	WB

Table S3. Primers used for qRT-PCR

Gene	Accession number	Primer sequences	Annealing temperature (°C)	Product size (bp)
β-actin	NM_001101.4	Forward: 5’-AGAGCTACGAGCTGCCTGAC-3’<br>Reverse: 5’-AGCACTGTGTTGGCGTACAG-3’	60	184
Nanog	NM_024865.3	Forward: 5’-TGTGTTCTCTTCCACCCAGC-3’<br>Reverse: 5’-CTTCTGCGTCACACCATTGC-3’	60	205
Oct4	NM_002701.5	Forward: 5’-ATGTGGTCCGAGTGTGGTTC-3’<br>Reverse: 5’-ACAGTGCAGTGAAGTGAGGG-3’	60	186
Sox2	NM_003106.3	Forward: 5’-ATGGGTTCGGTGGTCAAGTC-3’<br>Reverse: 5’-ACATGTGAAGTCTGCTGGGG-3’	60	166
SCDAL	ENST00000617440.1	Forward: 5’-GGAAACGTGGCTGTAGGACA-3’<br>Reverse: 5’-GGGGAAGGTGAAGGAGAAGC-3’	60	224
ACKR3	NM_020311.2	Forward: 5’-ACGTGGTGGTCTTCCTTGTC-3’<br>Reverse: 5’-AAGGCCTTCATCAGCTCGTA-3’	60	220
CXCL12	NM_000609.6	Forward: 5’-TCAGCCTGAGCTACAGATGC-3’<br>Reverse: 5’-CTTTAGCTTCGGGTCAATGC-3’	60	161
GDF6	NM_001001557.3	Forward: 5’-TGCACGTGAACTTCAAGGAG-3’<br>Reverse: 5’-CCCGCGTCGATGTATAGAAT-3’	60	232
MMP9	NM_004994.2	Forward: 5’-TTGACAGCGACAAGAAGTGG-3’<br>Reverse: 5’-GCCATTCACGTCGTCCTTAT-3’	60	179
MMP1	NM_002421.3	Forward: 5’-GGTCTCTGAGGGTCAAGCAG-3’<br>Reverse: 5’-AGTTCATGAGCTGCAACACG-3’	60	207
MMP23B	NM_006983.1	Forward: 5’-CCTCCGGATAGGCTTCTACC-3’<br>Reverse: 5’-CAGGACCCAGTACTCGCTGT-3’	60	151

BAMBI	NM_012342.2	Forward: 5’-GCCTCAGGACAAGGAAACAG-3’<br>Reverse: 5’-CCGTGAAAGCTGTAGTGCAA-3’	60	242
CRIM1	NM_016441.2	Forward: 5’-GGAAGGAGAAACGTGGAACA-3’<br>Reverse: 5’-GTCAGGCTTCCAGGACTCAG-3’	60	247
ACVRL1	NM_000020.2	Forward: 5’-ATTACCTGGACATCGGCAAC-3’<br>Reverse: 5’-TTGGGCACCACATCATAGAA-3’	60	214
WNT5B	NM_030775.2	Forward: 5’-GTGCAGAGACCCGAGATGTT-3’<br>Reverse: 5’-GTCTCTCGGCTGCCTATCTG-3’	60	242
WLS	NM_024911.6	Forward: 5’-GCCAGCTATGAGCAAAGTCC-3’<br>Reverse: 5’-TGGGATGGTGCATACAAGAA-3’	60	246
DKK1	NM_012242.3	Forward: 5’-TCCGAGGAGAAATTGAGGAA-3’<br>Reverse: 5’-CCTGAGGCACAGTCTGATGA-3’	60	157
EPOR	NM_000121.3	Forward: 5’-GAGCATGCCCAGGATACCTA-3’<br>Reverse: 5’-TACTCAAAGCTGGCAGCAGA-3’	60	194
NEK7	NM_133494.2	Forward: 5’-ATTGGTCGCGGACAATTTAG-3’<br>Reverse: 5’-GTCGCCAGCATCTGCTAGTT-3’	60	237
CDKN1C	NM_000076.2	Forward: 5’-CACGATGGAGCGTCTTGTC-3’<br>Reverse: 5’-CCTGCTGGAAGTCGTAATCC-3’	60	173
CDCA4	NM_017955.3	Forward: 5’-TGTTTGCACGAGGACTGAAG-3’<br>Reverse: 5’-TGTTGGCAATGAGGACTGAG-3’	60	192
PTX3	NM_002852.3	Forward: 5’-GTGGGTGGAGAGGAGAACAA-3’<br>Reverse: 5’-TTCCTCCCTCAGGAACAATG-3’	60	175
CLDN11	NM_005602.5	Forward: 5’-CTGGTGGACATCCTCATCCT-3’<br>Reverse: 5’-CCAGCAGAATGAGCAAAACA-3’	60	190
NR2F2	NM_021005.3	Forward: 5’-CAACTGCCACTCGTACCTGT-3’<br>Reverse: 5’-CAACACAAACAGCTCGCTCC-3’	60	250
Mouse β-actin	XM_030254057.1	Forward: 5’-AGATCAAGATCATTGCTCCTCCT-3’<br>Reverse: 5’-ACGCAGCTCAGTAACAGTCC-3’	60	174

Table S4. Primers used for RACE, RIP and ChIP

	Name	Forward primer (5’-3’)	Reverse primer (5’-3’)
**RACE**	3’ RACE nest round 1	CAGCCCCTTGGAAGGGCTCAGAT	GCTGTCAACGATACGCTACGTAAC

	3’ RACE nest round 2	CCATCCTCCTGGGGCTCCCTAGAC GCTGTCAACGATACGCTACGTAAC	GCTACGTAACGGCATGACAGTG
	5’ RACE nest round 1	GGCATGACAGTGCCCCCCCCCCCC CCC	CCACTTTCGCGCCTTCCATCATCT
	5’ RACE nest round 2	GCTGTCAACGATACGCTACGTAAC	GCCTGGGAACTTGCCCTGGACTT
	1-865	GACTCTTGGAGTGTAAATCTTATC GTC	TCTTGGTAAAGAACACTTCCGCTA
	665-1500	AGTTCGGAGCAATTGGAATCA	TATTCTCTCTGCCAGGGAACAA
	1358-2287	CCTCCCTTCTCTTTCTGGTTTG	ACTAAGAAGGGCACGATGGC
	2119-2915	CTCTGTTTCCTCCTCTCAGCTTG	CCATTTAAAAGCCCTTTATTTAAA A
**RIP**	*SCDAL* 23-354	TCGTCCCTTCTGATAACACACT	AGGAACGTCATTTCGAGGGG
	SCDAL 335-733	CCCCTCGAAATGACGTTCCT	TCAGCTTCAACACACGCTCT
	SCDAL 714-1126	AGAGCGTGTGTTGAAGCTGA	TCCATCATCTCGTGCCTGGG
	SCDAL 1108-1557	CCAGGCACGAGATGATGGAA	GTTCCTGGGGCTTCCCTTC
	SCDAL 1539-1921	GAAGGGAAGCCCCAGGAACT	GAAGGCGAATCCCCGTTGA
	SCDAL 1925-2375	CCAGCGAGGGAGGTTTACTC	AGCCACGATCCCAGACACTA
	SCDAL 2355-2771	TTAGTGTCTGGGATCGTGGC	TCCTCCCCCTCAAAAGGAGA
	SCDAL 2566-2889	GCAGGGGCAAAACTTCCATATC	GAACAAAGATAACTGACAAGCGA
**ChIP**	GDF6 promoter 113-467nt up TSS	TCCAAGCTGTCTCTTGGGGG	TGGGGAGGGTGGGAGGAGAG
	GDF6 promoter 448-796nt up TSS	CTGCGTCAGAAGAGGTTGGG	CCCCCAAGAGACAGCTTGGA
	GDF6 promoter 775-1073nt up TSS	ATTGGGGTAGAGCTGCAAAGG	TCCCCAACCTCTTCTGACGC
	GDF6 promoter 1056-1443nt up TSS	CTGTCCCTTGCTGCATAACC	TTGCAGCTCTACCCCAATCTG
	GDF6 promoter 1409-1780nt up TSS	GTGGAGGGCATATCCCAGAC	GCCACTCTGCCAAGAGGTTA
	GDF6 promoter 1757-2154nt up TSS	TTAAAGCACAGAGAGAGGAAGG	TAAGGTCTGGGATATGCCCTC
	GDF6 promoter 2128-2482nt up TSS	ATGGGGAGTTTTGAGTCTGTG	TTCTTCCTTCCTCTCTCTGTGCT

Table S5. Sequences for Ribo™ lncRNA Smart Silencer

	Target sequences	Sense (5’-3’)	Antisense (5’-3’)
SCDAL-ASO-1	CTCAGCTTGAGGCTCTCCTG	CAGGAGAGCCTCAAGC TGAG	
SCDAL-ASO-2	GTTGCCAGGAGACATCAGT G	CACTGATGTCTCCTGGC AAC	
SCDAL-ASO-3	CTGTGATCCTTACCGCACAG	CTGTGCGGTAAGGATC ACAG	
SCDAL-siRNA-1	GCTACGAATGTAAGATGAA	GCUACGAAUGUAAGAU GAAdTdT	dTdTCGAUGCUUACAUUC UACUU
SCDAL-siRNA-2	CAGAGATACCTGTTATCAA	CAGAGAUACCUGUUAU CAAdTdT	dTdTGUCUCUAUGGACAA UAGUU
SCDAL-siRNA-3	GTACTTCGCTTGTCAGTTA	GUACUUCGCUUGUCAG UUAdTdT	dTdTCAUGAAGCGAACAG UCAAU

Table S6. Sequences for siRNAs

siRNAs	Sense (5’-3’)	Antisense (5’-3’)
GDF6	GCUAAUACGAUCACCAGCUTT	AGCUGGUGAUCGUAUUAGCTT
SNF5	GCAACGAUGAGAAGUACAATT	UUGUACUUCUCAUCGUUGCTT

References

[1] R. Wu, B. Gu, X. Zhao, Z. Tan, L. Chen, J. Zhu, M. Zhang, *Hum Cell* **2013**,

26, 19.

[2] a) X. Hu, R. Wu, Z. Jiang, L. Wang, P. Chen, L. Zhang, L. Yang, Y. Wu, H. Chen, H. Chen, Y. Xu, Y. Zhou, X. Huang, K. A. Webster, H. Yu, J. Wang, *Stem cells (Dayton, Ohio)* **2014**, 32, 2702; b) F. Yang, R. Wu, Z. Jiang, J. Chen, J. Nan, S. Su, N. Zhang, C. Wang, J. Zhao, C. Ni, Y. Wang, W. Hu, Z. Zeng, K. Zhu, X. Liu, X. Hu, W. Zhu, H. Yu, J. Huang, J. Wang, *Cell death & disease* **2018**, 9, 556.

[3] D. Kim, G. Pertea, C. Trapnell, H. Pimentel, R. Kelley, S. L. Salzberg, *Genome Biol* **2013**, 14, R36.

[4] M. Guttman, M. Garber, J. Z. Levin, J. Donaghey, J. Robinson, X. Adiconis, L. Fan, M. J. Koziol, A. Gnirke, C. Nusbaum, J. L. Rinn, E. S. Lander, A. Regev, *Nature biotechnology* **2010**, 28, 503.

[5] C. Trapnell, B. A. Williams, G. Pertea, A. Mortazavi, G. Kwan, M. J. van Baren, S. L. Salzberg, B. J. Wold, L. Pachter, *Nature biotechnology* **2010**, 28, 511.

[6] L. Sun, H. Luo, D. Bu, G. Zhao, K. Yu, C. Zhang, Y. Liu, R. Chen, Y. Zhao, *Nucleic Acids Res* **2013**, 41, e166.

[7] L. Kong, Y. Zhang, Z. Q. Ye, X. Q. Liu, S. Q. Zhao, L. Wei, G. Gao, *Nucleic Acids Res* **2007**, 35, W345.

[8] A. Bateman, E. Birney, L. Cerruti, R. Durbin, L. Etwiller, S. R. Eddy, S. Griffiths-Jones, K. L. Howe, M. Marshall, E. L. Sonnhammer, *Nucleic Acids Res* **2002**, 30, 276.

[9] M. F. Lin, I. Jungreis, M. Kellis, *Bioinformatics* **2011**, 27, i275.

[10] M. Heiss, M. Hellstrom, M. Kalen, T. May, H. Weber, M. Hecker, H. G. Augustin, T. Korff, *FASEB journal : official publication of the Federation of American Societies for Experimental Biology* **2015**, 29, 3076.

[11] S. Konermann, M. D. Brigham, A. E. Trevino, J. Joung, O. O. Abudayyeh, C. Barcena, P. D. Hsu, N. Habib, J. S. Gootenberg, H. Nishimasu, O. Nureki, F. Zhang, *Nature* **2015**, 517, 583.

[12] D. Kim, B. Langmead, S. L. Salzberg, *Nat Methods* **2015**, 12, 357.

[13] M. Pertea, G. M. Pertea, C. M. Antonescu, T. C. Chang, J. T. Mendell, S. L. Salzberg, *Nature biotechnology* **2015**, 33, 290.

[14] Y. Liao, G. K. Smyth, W. Shi, *Bioinformatics* **2014**, 30, 923.

[15] S. Anders, W. Huber, *Genome Biol* **2010**, 11, R106.

[16] a) N. Jiang, J. Huang, L. J. Edwards, B. Liu, Y. Zhang, C. D. Beal, B. D. Evavold, C. Zhu, *Immunity* **2011**, 34, 13; b) J. Hong, S. P. Persaud, S. Horvath, P. M. Allen, B. D. Evavold, C. Zhu, *Journal of immunology (Baltimore, Md. : 1950)* **2015**, 195, 3557; c) V. I. Zarnitsyna, C. Zhu, *Journal of visualized experiments : JoVE* **2011**, DOI: 10.3791/3519e3519.

[17] C. Xiao, K. Wang, Y. Xu, H. Hu, N. Zhang, Y. Wang, Z. Zhong, J. Zhao, Q. Li, D. Zhu, C. Ke, S. Zhong, X. Wu, H. Yu, W. Zhu, J. Chen, J. Zhang, J. Wang, X. Hu, *Circulation research* **2018**, 123, 564.
